# Children adjust behavior in novel social environment to reflect local prosocial norms inferred from brief exposure

**DOI:** 10.1371/journal.pone.0325984

**Published:** 2025-07-09

**Authors:** Kari Britt Schroeder, Laura Nelson Darling, Peter R. Blake

**Affiliations:** Department of Psychological and Brain Sciences, Boston University, Boston, Massachusetts, United States of America; University of Nevada Reno, UNITED STATES OF AMERICA

## Abstract

Stark cultural variation in prosocial behavior, as elicited with economic experiments, is evident despite the high mobility of humans. Conformity to local norms has been posited to play an integral role in the maintenance of this variation. Experiments suggest that adults indeed rapidly infer pro- and antisocial norms in new or altered social environments and adjust their behavior to reflect the inferred norms. Studies of the ontogeny of prosocial behavior show that by middle childhood, children’s prosocial behavior conforms to that of local adults. Furthermore, by this stage, children are susceptible to the manipulation of explicit normative information. However, their propensity to extract or infer normative information from the environment and change their behavior accordingly has not been investigated. Here, we assess whether children 1) rapidly infer local prosocial norms in a novel, realistic social environment, 2) extend these inferences to norms for unobserved behaviors, and 3) alter their behavior in the novel environment to align with the inferred norms while still 4) maintaining their baseline prosocial behavior outside of the novel environment. We used questionnaires to measure children’s perceived pro- and antisocial descriptive norms in their Own Neighborhoods as well as in a novel “Neighborhood X,” to which they were introduced via a slideshow. Norms for Neighborhood X diverged drastically dependent upon which slideshow they witnessed (Prosocial or Antisocial condition), a result robust to the exclusion of questions about norms for behaviors observed in the slideshow. Children’s perceptions of prosocial norms in their Own Neighborhoods predicted their prosocial behavior (Dictator Game) in their Own Neighborhood. Moreover, even though information about giving behavior was not presented in the slideshow, inferred norms for Neighborhood X predicted children’s prosocial behavior in that neighborhood as well. These changes in prosocial behavior were transitory and specific to Neighborhood X; prosocial behavior in a separate “Helping Task” was best predicted by prosocial norms within the children’s Own Neighborhoods. Our results are consistent with the hypothesis that humans have a propensity to rapidly infer and conform to local prosocial norms, thus maintaining group differences in prosocial behavior, and further indicate that this propensity is in operation by middle childhood.

## Introduction

Humans are remarkable in their capacity to behave prosocially towards non-kin. Prosocial behaviors include acts that are costly to the individual and deliver a benefit to others. We define prosociality broadly to include helping and giving (i.e., altruism) as well as cooperative actions that are mutualistic or beneficial to one’s group [1]. Whereas donating blood and relinquishing one’s seat on a bus are examples of altruistic behaviors, cooperative behaviors include shoveling the snow from the sidewalk in front of one’s house, participating in a Neighborhood Watch program, and recycling [2]. Because they benefit others, prosocial behaviors are often moralized as “good;” prosociality itself has been described as a multifaceted construct that includes internalized morality [3].

Pronounced cultural differences in prosocial behavior [4–6], already apparent by middle childhood [7–10], have been revealed with experimental economic games such as the Dictator Game and Public Goods Game. This persistent cultural variation in prosocial behavior is surprising given how mobile humans are [11,12]. A fine-scale example of this phenomenon was illustrated in a longitudinal study by Smith et al. [13], who tracked the movement of Hadza hunter-gatherers from camp to camp while also gathering data on cooperation, as elicited by experimental contributions to the public good. Despite substantial residential mixing, inter-camp variation in contributions to the public good was maintained; individual contributions were best predicted by the mean contribution of the other camp members and not by an individual’s previous contribution.

Such behavioral plasticity is consistent with evolutionary models of prosocial behavior that emphasize the primacy of social norms (i.e., informal standards of behavior shared by a group) [13]. The “norm psychology” hypothesis of Chudek and Henrich [14] proposes that humans possess a suite of cognitive mechanisms and motivations that enable the inference, adoption, and enforcement of social norms. In support of this hypothesis, a wealth of research shows that descriptive norms (i.e., norms that describe what most people actually do, as opposed to injunctive norms, which identify what is considered appropriate behavior [15]) play an important role in human behavior, from voting [16], to alcohol [17] and fruit [18] consumption, to driving violations [19]. Furthermore, the effect of descriptive norms extends to pro- or antisocial behavior. For example, field studies in which adults were given explicit written information about others’ prosocial behavior demonstrated an increased compliance with prosocial norms of sustainability (higher rate of towel reuse at a hotel [20,21] or limited energy consumption [22]) or of charitable giving [23]. In the lab, participants in a Dictator Game (DG; the “dictator” is given an endowment and decides how much, if any of it to transfer to the “recipient”) who were told about the generosity of prior players (real or imagined) were more generous in their choices than those who were told about the selfishness of prior players [24,25].

Importantly, additional field and lab studies show an effect of descriptive norms on pro- and antisocial behavior even when normative information is not explicitly given to the participant but must be inferred from the social environment or observed behavior of others. In a seminal series of field studies, Cialdini, Reno, and Kallgren [15] manipulated the amount of litter in a parking garage, which was sufficient to induce changes in the rate of littering. Other field and lab studies have found that adults who are exposed to implicit pro- or antisocial normative information will generalize that information to other, similarly-valenced behaviors and subsequently apply the inferred norm. For example, Keizer, Lindberg, and Steg [26] demonstrated what they term a “cross-norm effect;” that is, environmental evidence of antisocial norms induced an increase in *other* antisocial behaviors (e.g., graffiti adjacent to a sign prohibiting graffiti led to a higher rate of littering in the same location); similarly, participants who thought that their neighbors were more likely to cheat on taxes, benefits, or public transport fares took more money from an anonymous neighbor in an experimental economic game [27]. Such a cross-norm effect has also been observed with prosocial behavior; brief exposure to cues of prosocial norm adherence in a naturalistic setting was associated with an increased rate of adherence to other prosocial norms [28]. This phenomenon has also been observed in the lab, where experience with either a pro- or antisocial “culture,” via an experimental economic game, resulted in divergent levels of general trust and prosocial behavior in subsequent economic games [29].

The studies sketched above demonstrate that adults have the propensity to 1) rapidly infer local pro- and antisocial norms through observation of a realistic social environment, 2) extend these inferences to norms for unobserved behaviors or novel situations (the “cross-norm effect”), and 3) alter their behavior in alignment with the inferred norms. Furthermore, we suggest that 4) any behavioral changes should be specific to the novel social environment (i.e., norms are specific to a place or institution). We argue that these four capabilities constitute critical components of human norm psychology that facilitate behavioral plasticity. Combined, they enable a newcomer to rapidly approximate local normative behavior in a novel environment—be it an unfamiliar place or institution—without either being given explicit normative information or spending years witnessing a multitude of different social situations.

### The ontogeny of normative conformity

Despite the potential importance of behavioral plasticity in the maintenance of among-group variation in prosocial behavior, the ontogeny of these capabilities has not been well-established. Some evidence may be found in studies that show that the transmission of norms of sharing occurs not just through teaching but via observation and imitation [30–34]. Imitation may also be implicated in the development of reciprocity [35]. That is, while preschool-age children engage in both downstream and upstream positive indirect reciprocity—i.e., they are more likely to behave prosocially towards a peer who has just treated another child prosocially [36] or towards a third person after being treated prosocially themselves [37]—they do not necessarily discriminate between direct and indirect positive reciprocity [35,38].

Furthermore, there is clear evidence of plasticity in prosocial behavior in children. Evidence for the long-term malleability of prosocial behavior comes from intervention studies. For example, children assigned to a mentoring program during middle childhood showed increased prosociality relative to a control group, ostensibly due to more intense social interactions and attachment to a prosocial figure; this increase in prosociality was still evident two years later at the beginning of adolescence [39]. Similarly, children who were randomized into a preschool exhibited more egalitarian preferences in middle childhood then those where not [40].

Evidence for the short-term plasticity of prosocial behavior in children—and the salience of prosocial norms for behavioral plasticity—comes largely from DG studies. The DG has been widely used with children, with the robust finding that prosociality increases with age [10,41]; both positive and negative effects of socioeconomic status (SES) on DG behavior in children have been observed [7,10,41–43]. Children’s behavior in the DG children mirrors that of local adults, i.e., exhibits similar cultural differences, by around middle childhood [8]. Recent work demonstrates that this coincides developmentally with increasing responsiveness to the experimental manipulation of normative information. In a study including six diverse societies, House et al. [44] exposed children aged four to fifteen to an injunctive norm manipulation before they engaged in a DG. The manipulation consisted of a video recording of a local adult saying that the generous choice (selfish choice) in the DG was “right” and “good to choose” (“wrong” and “bad to choose”); for the control, both options were “OK to choose.” Children in all six societies responded reliably, choosing the generous option more often after the injunctive manipulation relative to the control. These effects emerged between six and eight years of age and increased in strength across childhood, such that by ten years of age, children’s behavior was comparable to that of local adults. Similar results were obtained in a separate study with a more limited (German) sample [45].

In another norm manipulation DG study with children, McAuliffe, Raihani, and Dunham [46] primed children in the U.S. aged four to nine years with either a generous or a selfish norm. The experimenter told the child either how many candies they thought the child should give to the recipient (injunctive norm) or how many candies they thought most other children who play the game give to the recipient when playing the game (descriptive norm). Although children primed with a generous norm gave more than those who were primed with a selfish one (irrespective of whether it was injunctive or descriptive), the authors did not observe a clear developmental trajectory.

Recent studies have moved beyond the explicit verbal manipulation of norms to look at the effects of a group majority on prosocial behavior. In a series of DG experiments, Misch and Dunham [47] and Chai et al. [48] showed that children aged four to eight years behaved more prosocially in a DG after observing a video of two to three adults or peers model prosocial DG behavior (descriptive norm); for younger children, observation of in-group peers modeling antisocial DG behavior also resulted in markedly antisocial DG behavior [47]. That this result was not observed when a single person repeated the behavior multiple times speaks to a normative, rather than purely imitative, interpretation [48].

In sum, the studies outlined above indicate that observation and imitation play a role in the development of prosocial behavior, that prosocial behavior is malleable in children, and that children adjust their prosocial behavior to conform to explicit and specific normative information by middle childhood. Moreover, the results of House et al. [44,45] suggest that the developmental emergence of group differences in prosocial behavior around middle childhood is precipitated, at least in part, by increasing responsiveness to local prosocial norms. What has not yet been established, however, is whether and how children in middle childhood adapt to new social environments. Building on the norm psychology account, we hypothesized that children would: 1) rapidly infer pro- and antisocial norms through observation of a realistic novel social environment, 2) extend these inferences to norms for unobserved behaviors, and 3) adjust their behavior to be in alignment with the inferred norms—but 4) only within the novel social environment.

### Current study

We used a novel approach to determine whether children in middle childhood engage in norm inference and behavioral plasticity when exposed to a new neighborhood. We first assessed the children’s perceptions of descriptive norms (both pro- and antisocial) in their Own Neighborhoods and had them play a DG with a child from their Own Neighborhood. This provided a baseline for children’s experience and prosocial behavior. We next used a slideshow to introduce children to a novel neighborhood, which we called “Neighborhood X.” Children were randomly assigned to view either a Prosocial or Antisocial version of the neighborhood (Neighborhood condition), depicted in images of closely matched behaviors and descriptions (e.g., someone either spray-painting or cleaning up graffiti). After the neighborhood “tour,” we assessed the children’s perceptions of descriptive norms (both pro- and antisocial) in Neighborhood X and had them play a DG with a child from Neighborhood X.

The descriptive norm assessment consisted of a questionnaire with Likert-type items referring to the frequency of five behaviors such as recycling or littering (we refer to these behaviors as either *positive* or *negative* to distinguish them from condition). Critically, children did not observe three of these five behaviors in the slideshow of Neighborhood X. This allowed us to test whether children extended their inferences about prosocial norms to unobserved behaviors.

In addition to the descriptive norm assessment, we included a personal (as opposed to social) [49,50] injunctive norm assessment for the same five behaviors for the child’s Own neighborhood and Neighborhood X. This provided a check to ensure that children’s personal beliefs about the acceptability of antisocial behaviors had not changed due to the neighborhood manipulation.

Lastly, to evaluate the specificity of any changes in prosocial behavior following exposure to Neighborhood X, we gave the children the option to help us (the experimenters) by engaging in a real-effort encryption task [51] (Helping Task) at the end of the study. Finally, given a well-established effect of positive emotions on prosocial behavior [52], we included a measure of affect in order to assess whether expected condition-dependent differences in DG behavior could be attributed solely to differences in affect arising from the slideshows.

### Predictions

Following the norm psychology account, we predicted that children 1) would infer that the Prosocial Neighborhood X had more prosocial descriptive norms than the Antisocial Neighborhood X and 2) would extend their judgments of descriptive norms to unobserved behaviors. We also predicted that 3a) children would adjust their behavior to Neighborhood X, giving more in a DG in the Prosocial Neighborhood condition compared to the Antisocial Neighborhood condition and that 3b) within each neighborhood (children’s Own Neighborhood and Neighborhood X), more prosocial norms would predict higher donations. Lastly, we anticipated that 4) any change in children’s prosocial behavior due to neighborhood condition (Prosocial or Antisocial Neighborhood X) would not extend beyond that neighborhood. More specifically, we did not expect an effect of condition or perceived norms in Neighborhood X on the final Helping Task (helping the experimenter).

## Materials and methods

### Ethics statement

The study was pre-approved by the Boston University Institutional Review Board (Protocol #3501E), and informed consent was given by a parent or guardian of all participants. This study was conducted in 2014 and 2015, before preregistration became prevalent [53], and thus we did not preregister our research plans.

### Participants

We recruited children aged nine to eleven years from the Boston area via emails and phone calls to families in a participant database maintained by the Boston University Social Development and Learning Lab (see S1 Appendix for exclusion of children based on certain criteria). Our final dataset, following data cleaning (S1 Appendix), includes 99 children (49 in the Antisocial condition, of which 25 are girls, and 50 in the Prosocial condition, of which 25 are girls). The mean age of participants was 9.9 years (34 nine-year-olds, 43 ten-year-olds, and 22 eleven-year-olds).

Parents completed an online survey, allowing us to collect demographic information about the child (e.g., age, race, etc.) and the neighborhood in which they lived (e.g., how long the family had lived there and in what type of dwelling) (S1 Appendix).

The participants all lived in the Boston area, with the majority residing in Middlesex and Suffolk counties, where Cambridge and Boston are located. All parents confirmed that their participating children had not received a diagnosis of autism or dyslexia and were not taking any psychoactive drugs (S1 Appendix). Ninety-five of the 99 participants were born to parents who both considered themselves of European ancestry. (We limited participation in this manner in order to maximize the likelihood that participants would make inferences based upon the behavior of the actors in the slideshow, who were white, rather than focusing on group membership based on race or ethnicity.) All but one of the participants resided with both of their parents. The majority of the participants resided in homes or apartments owned by their parent(s) (87.9%), indicating high SES, and had resided in the same neighborhood since infancy (56.6%). Only 15.2% of participants had lived in their neighborhoods for less than five years; of these, only three had moved neighborhoods more than once in the past five years.

### Protocol

**Fig 1:**
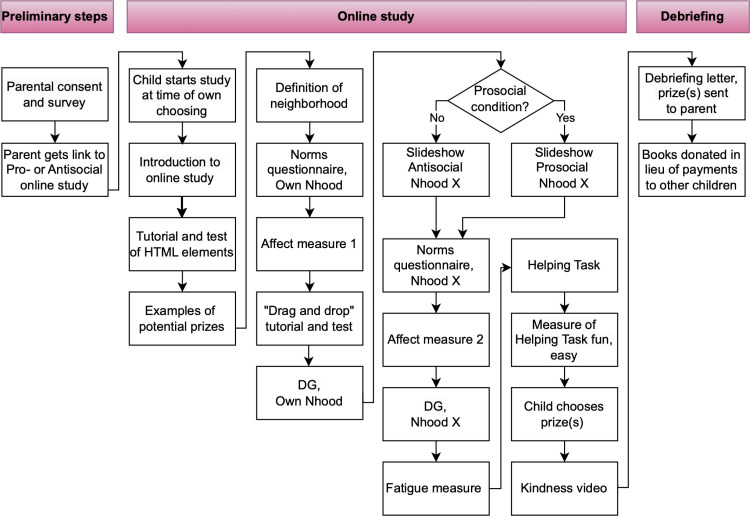
Flow chart of study protocol.

The study protocol is visualized in Fig 1. Once we had received informed consent from the parent, he or she received a child assent form to go over with their child. We randomly assigned each child to the Prosocial or Antisocial Neighborhood condition, and parents were given a condition-specific link to the online platform (Qualtrics), allowing the child to commence the study at the time of their choosing. Parents were instructed to let the child complete the study by themselves in one sitting (expectation of 40–55 minutes), at a laptop or desktop computer, with minimal distractions (e.g., TV, other online activities, other people in the room). Parents were informed that their child could stop participating at any time and still get a prize.

Once online, participants were given an expectation of the duration of the study and told that it was important that they complete the study by themselves and not have anyone looking over their shoulder. They were also informed that they could stop the study at any time and still receive a prize. They then received tutorials on the use of radio buttons, text boxes, drag and drop, and the Qualtrics Heat Map feature, which allows users to indicate certain points on an image. These tutorials were followed by checks for comprehension and participant engagement (S1 Appendix) as well as examples of potential prizes the participants could receive; they were told that the kind and number of prizes of prizes available to them would depend on the choices they made during the study.

Children received the following definition of neighborhood as: “the area where you live. It includes people who live near you. These are your neighbors. You might talk to or play with your neighbors. Or you might not talk to or play with your neighbors. Your neighborhood might also include places near where you live. Maybe there is a park, a library, or a store in your neighborhood that you visit.” They were told that if they lived in multiple homes in different neighborhoods, when they were asked questions about their neighborhood, they were to consider the one in which they spent the most time. The remainder of the study progressed as depicted in Fig 1 and as follows.

#### Own Neighborhood descriptive norms.

We asked participants about descriptive norms for five behaviors. The five behaviors (recycling, helping an elderly neighbor carry groceries, littering, keeping a library book forever, keeping a package that was supposed to be delivered to another household) can be performed by both children and adults and, collectively, represent both positive and negative behaviors directed towards both individuals and the community. For descriptive norm questions, we asked about both child and adult actors. For example, we asked about recycling as follows: “Do you think many adults (kids) in your neighborhood would Recycle?” A five-point scale was anchored at “No One Would” and “Everyone Would,” with the intermediate choices of “A Few Would,” “About Half Would,” and “Most Would.”

#### Personal injunctive norms before viewing Neighborhood X.

After asking about descriptive norms, we asked children about their personal injunctive norms, i.e., how good or bad they believed each behavior to be. These questions were not location or actor-specific. We asked about the personal injunctive norm for recycling as follows: “What do you think about Recycling? Do you think it’s Never OK to Recycle, Always OK to Recycle, or somewhere in between?” A five-point scale was anchored at “Never OK” and “Always OK,” with the intermediate choices of “Sometimes OK,” “OK About Half the Time,” and “Usually OK.”

#### Affect before viewing Neighborhood X.

Participants completed a self-assessment of the valence of their current emotional state, using a seven-point smiley/frowny face scale.

#### Own Neighborhood Dictator Game.

After completing the Own Neighborhood norm questionnaire and self-assessment of affect, children were introduced to a “Quarter Game” created using the drag and drop feature in Qualtrics. Participants were shown 13 quarters and told that they could divide them between themselves and a child in “Your Neighborhood.” Children were told that this recipient child would never know their name, and that they would never know who the other child was either (see S1 Appendix for script). After answering comprehension questions for the task (S1 Appendix), they moved each of 13 quarters (digital images) to one of two boxes, “Other Child’s Box” or “Your Box.” They also entered, in text boxes, the number of quarters they and the Other Child would receive.

#### Neighborhood X.

After the Own Neighborhood phase, children were given a slideshow tour of a new neighborhood, Neighborhood X. Prior to the slideshow, participants were told that we, the researchers, had been studying a neighborhood, “Neighborhood X,” which was near Boston but probably not home to anyone they know. We had been watching the people who lived there and taking notes on them and we wanted to share what we had seen, after which we would ask them questions about Neighborhood X.

**Fig 2:**
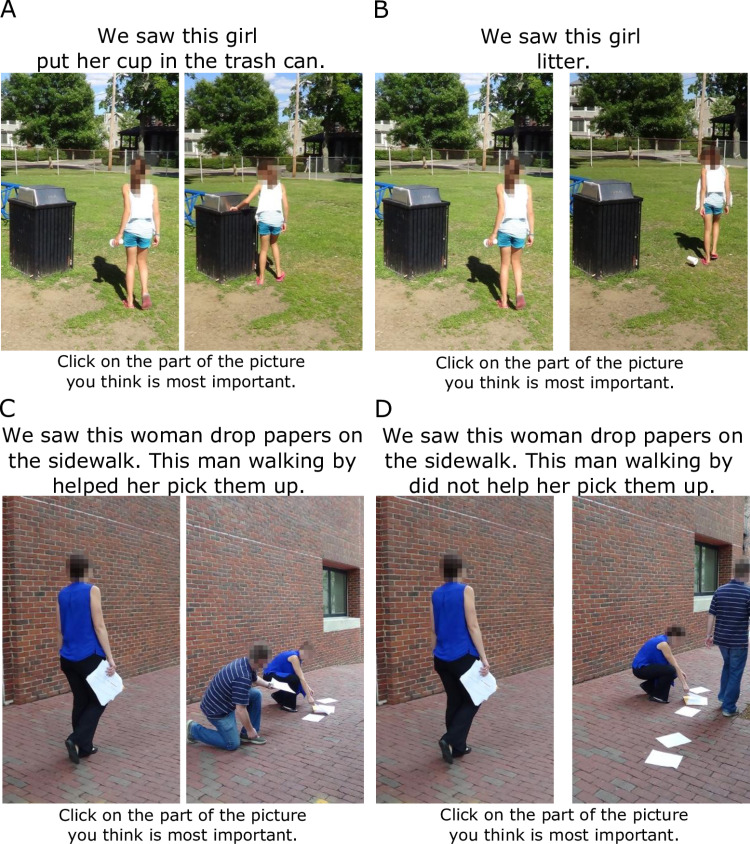
Examples of stimuli used to introduce children to Neighborhood X. Photographs and text from the “litter” (A is Prosocial condition, B Antisocial) and “dropped papers” (C is Prosocial, D Antisocial) stimuli (see Table 1). Note that for Fig 2 we have increased the size of the text relative to the image (approximately 200% increase compared to ratio used in slideshow stimuli).

The photos and descriptions of Neighborhood X varied depending on whether children had been assigned to the Prosocial or Antisocial Neighborhood condition. We used the same actors and situations in each condition, matching the positive and negative behaviors as closely as possible. We set the following criteria for these situations (Fig 2 and Table 1): 1) representation with one or two photos, 2) no physical harm, and 3) symmetry of people and setting for both conditions. Creation and validation of the images and text in the slideshow is treated in the S1 Appendix. The situations and behaviors for the Prosocial and Antisocial conditions are listed in Table 1.

**Table 1:** Description of photographic stimuli used in Neighborhood X slideshow. *N* and *Gender* refer to the number of actors in the image and their gender.

Stimulus	Prosocial Stimulus Text	Antisocial Stimulus Text	N	Gender
lost letter	We saw that someone had dropped their mail next to the mailbox. We saw this woman pick it up and put it in the mailbox.	We saw that someone had dropped their mail next to the mailbox. We saw this woman look at it and keep walking.	1	F
library book	We saw this girl put library books back on the shelf after she was finished with them.	We saw this girl ripping pages out of a library book.	1	F
slide queue	We saw this girl wait her turn to use the slide.	We saw this girl cut in line for the slide.	3	F
litter	We saw this girl put her cup in the trash can.	We saw this girl litter.	1	F
dropped cash	We saw this woman drop some money. Another woman picked it up and handed it back to her.	We saw this woman drop some money. Another woman picked it up and put it in her pocket.	2	F
chewed gum	We saw this boy take his gum out of his mouth and throw it away in a trash can.	We saw this boy take his gum out of his mouth and stick it on the bench.	1	M
door	We saw this man hold the door for the woman behind him. She was carrying a heavy package.	We saw this man who did not hold the door for the woman behind him. She was carrying a heavy package.	2	M, F
candy	We saw this woman take just one candy. The sign said to take one candy.	We saw this woman take a handful of candies. The sign said to take one candy.	1	F
dog poop	We saw this man clean up after his dog.	We saw this man who didn’t clean up after his dog.	1	M
dropped papers	We saw this woman drop papers on the sidewalk. This man walking by helped her pick them up.	We saw this woman drop papers on the sidewalk. This man walking by did not help her pick them up.	2	M, F
graffiti	We saw this woman cleaning up graffiti on a wall at the park.	We saw this woman spray painting a wall at the park with graffiti.	1	F

Participants progressed through the slideshow at their own pace. To maintain and gauge participant engagement, we used a feature of Qualtrics that enables tracking of user clicks; for each of the stimuli in the slideshow, participants were instructed to click on “the part of the picture you think is most important.”

#### Neighborhood X descriptive norms.

After the virtual tour of Neighborhood X, we asked participants about descriptive norms for the same five behaviors in that neighborhood.

#### Personal injunctive norms after viewing Neighborhood X.

We again asked children about their personal injunctive norms for each behavior to ensure that children’s own values had not changed due to exposure to the Antisocial neighborhood in particular.

#### Affect after viewing Neighborhood X.

Participants again completed a self-assessment of the valence of their current emotional state.

#### Neighborhood X Dictator Game.

After answering the norm questionnaire for Neighborhood X and a self-assessment of affect, children were introduced to a second “Quarter Game.” Participants were shown 13 quarters and were told that they could divide them between themselves and a child in “Neighborhood X.” The second DG otherwise proceeded exactly as the first, including comprehension questions. The participants were again told that the recipient child would never know their name and that they would never know who the other child was either (see S1 Appendix for script). For information on the distribution of the quarters given to the DG recipient in the child’s Own Neighborhood and in Neighborhood X, see below.

#### Helping task.

Participants were next given the opportunity to help the experimenters in a task. This consisted of a real-effort encryption task [51], in which participants used a new cipher to turn each four or five-letter word into a string of numbers. After each word, participants had the option to continue or stop, with the possibility of encrypting 20 words (they did not know how many encryption tasks there were in total). Fatigue and enjoyment were assessed on a five-point scale by asking participants how tired/energized they were prior to the Helping Task and how boring/fun the task was after completion.

#### Prize selection.

Participants chose one or more digital prizes from a selection of MP3s, games, and books they had viewed at the start of the experiment. The number and kind of available prizes depended on the number of quarters the participant had kept in the two DGs.

#### Kindness video.

At the end of the experiment, children watched a video on the internet about kindness among children.

#### Debriefing and distribution of DG allocations.

Parents received their child’s chosen prize(s) by email along with a debriefing letter, which disclosed the fabricated nature of the images, to read along with their child.

Because our IRB would not allow us to give out cash to children, we used the quarters participants had allocated to other children in the Own Neighborhood and Neighborhood X DGs to buy books. We collaborated with local libraries to identify and give over $264 in books to eight- to twelve-year-old children who lived in the two counties (Suffolk and Middlesex) in which we photographed the Neighborhood X stimuli and in which the majority of participants lived (based on zip code).

### Analyses

#### Regression analyses.

Our primary analyses consisted of investigating, via regression, the effect of neighborhood (Own or X) and condition (Antisocial or Prosocial) on responses to the descriptive norms questions and, subsequently, the effect of neighborhood, condition, and norms on the number of quarters given in the DGs. Because most of our outcome variables were ordinal (i.e., norms questions, the number of quarters given in the DGs, and affect before and after the slideshow), we primarily used ordered logit regression analysis. Many of our outcome variables were also repeated measurements (e.g., participants answered norms questions and played the DG twice, once for each neighborhood), thus introducing correlation between responses at the individual level, for which reason we also employed multilevel modeling.

For all regression analyses, we specified increasingly complex models in a stepwise fashion, assessing improvement in model fit with either the deviance information criterion (DIC) or Akaike Information Criterion [54]; depending on the outcome variable, we considered the following covariates: *age*, *gender* (boy), *years in neighborhood* (i.e., the number of years the participant had lived in their Own Neighborhood), *descriptive norms*, *Antisocial condition*, *Neighborhood X*, *adult* (descriptive norm question specific to adults, not children), *negative affect* (seven-point scale), *helping task fun* (five-point scale), and *energized* (five-point scale; asked prior to Helping Task).

For multilevel regression analyses, we specified models with varying intercepts for participants as well as behaviors (i.e., items in norm questionnaire); due to a substantial increase in variance in descriptive norm responses for Neighborhood X compared to Own Neighborhood (over 100%), we also allowed slopes for neighborhood (Own, X) to vary conditioned on participant and behavior. We also fit multilevel ordered logit regression models to a subset of the descriptive norms data for which there was no explicit information in the slideshow; i.e., we analyzed data for three of the five behaviors (recycling, helping an elderly neighbor carry groceries, and keeping a package that was supposed to be delivered to another household), excluding data for two behaviors (littering and keeping a library book forever). While we assessed models with varying intercepts for participants for this subset of the data (the norms questions were posed twice, for Own Neighborhood and Neighborhood X), we did not fit models with varying intercepts for behavior items or slopes for Neighborhood on behavior because of the reduced number of behavior items in the dataset. Rather, we created a fixed effect for the sole negative behavior (*negative behavior*).

We fit multilevel models in Rstan (Rstan [55] is the R [56] interface for Stan [57], a probabilistic programming language that implements Hamiltonian Monte Carlo sampling) using the R package glmer2stan [58], a convenience wrapper for Rstan, with weak priors for variance components (see glmer2stan [58] documentation) and initial values of zero for fixed effects and one for standard deviations. For initial model fitting, we ran four chains with at least 4,000 iterations each (half warm-up) and inspected trace plots to confirm convergence of the chains; for final estimates of reported models, we ran one chain, drawing between 10,000 and 12,000 samples from the posterior (half warm-up). We summarized parameter estimates and visualized model predictions from the fitted models using the R packages rethinking [59,60] and ggplot2 [61].

In some instances, for clarity of interpretation of the reported results, we also analyzed DG behavior in each neighborhood separately using a single-level ordered logit regression as implemented in the R package MASS [62]. Analysis of the number of completed Helping Tasks was conducted via Poisson regression.

For binomial and ordered logit regression analyses, we report Odds Ratios. An Odds Ratio, or OR, greater than one indicates that an increase in one unit of the predictor variable is associated with higher odds of, for example, a more prosocial response to the norms questions, or more quarters given in the DG. An OR between zero and one indicates that an increase in one unit of the predictor variable is associated with lower odds of a more prosocial response or more quarters given.

#### Prosocial norm indices.

To create composite prosocial norm indices from responses to the norm questions about five behaviors, we reverse-coded participant responses for negative behavior items, with one signifying least prosocial and five signifying most prosocial. Thus, for all results, an increase in prosocial norms indicates more prosocial and less antisocial norms. Because of minimal variation in responses to personal injunctive norms items, we collapsed injunctive responses across bins one through four, creating two categories.

Prosocial descriptive norm indices were derived by fitting a multilevel model without the covariate of *Antisocial condition* (Model 3 in Table 2). This allowed us to simultaneously estimate variation in responses due to both individual and item (i.e., specific behavior) covariates as well as changes in perceived norms across neighborhoods. Thus, as a measure of individual descriptive norms in Own Neighborhood, we used the mean estimate of participant intercepts. For changes in perceived norms between Own Neighborhood and Neighborhood X, we used the mean estimate of slopes for Neighborhood X, conditioned on participants. The sum of these values provided individual descriptive norms in Neighborhood X.

**Table 2:** Prosocial descriptive norms modeled as dependent upon neighborhood and condition. Ordered logit regression. The reference categories for the binary variables *Neighborhood X* and *Antisocial condition* are *Own Neighborhood* and *Prosocial condition*, respectively. Predictions derived from Model 2 are depicted in Fig 3. Parentheses contain 95% confidence intervals for ORs. Standard deviations given for estimated variance components. “Correlation” refers to correlation between estimated slopes, intercepts. Estimated cutpoints not included in table.

	*Model 1*			*Model 2*			*Model 3*		
	Est.	SD	OR	Est.	SD	OR	Est.	SD	OR
**Fixed effects**									
*Neighborhood X*	0.99	0.12	2.69 (2.12,3.42)	1.74	0.88	5.70 (2.32,14.73)	−1.14	0.57	0.32 (0.12,0.91)
*Antisocial condition*	0.23	0.12	1.26 (1.00,1.62)	0.34	0.28	1.40 (0.81,2.44)			
*Participant age*	−0.24	0.06	0.79 (0.70,0.88)	−0.30	0.18	0.74 (0.53,1.04)	−0.28	0.17	0.76 (0.54,1.06)
*Adult actor*	0.81	0.08	2.25 (1.90,2.64)	1.31	0.10	3.71 (3.03,4.48)	1.31	0.10	3.71 (3.00,4.53)
Neighborhood X * *Antisocial*	−3.62	0.19	0.03 (0.02,0.04)	−5.97	0.53	0.00 (0.00,0.01)			
**Variance components**									
*Participant intercepts*				6.79			1.22		
*Slopes for Nhood X* *on Participant*				4.43			4.02		
Correlation				−0.07			−0.29		
*Behavior Intercepts*				1.22			7.47		
*Slopes for Nhood X* *on Behavior*				2.33			1.05		
Correlation				−0.31			−0.06		
**DIC**	4760			3570			3572		

Additional R packages used include dplyr [63], lme4 [64], and reshape2 [65]. The data and code used to produce all figures and tables are found in S1 Dataset and S2 Appendix, respectively.

## Results

### Baseline prosocial norms and behavior in own neighborhood

**Fig 3:**
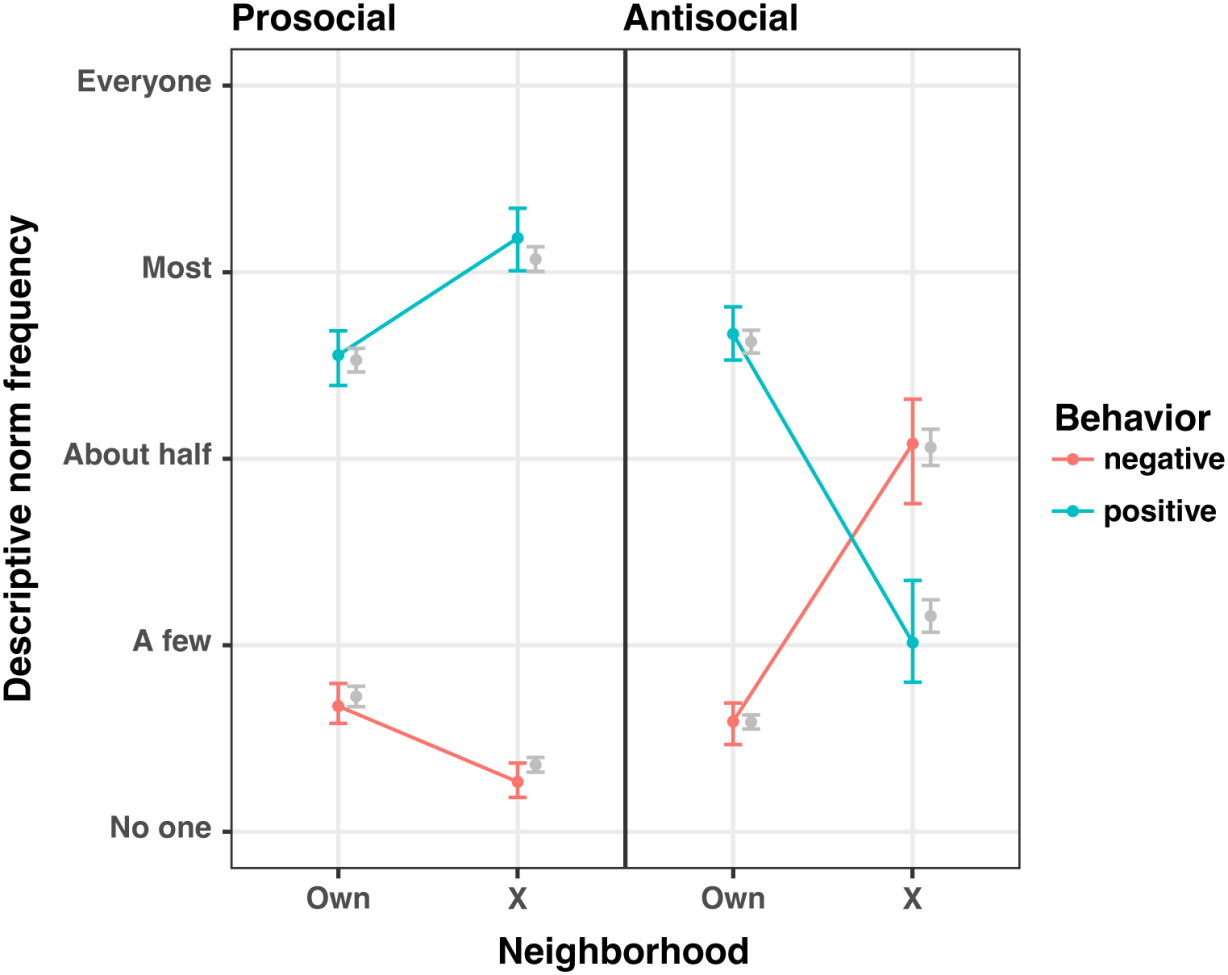
Descriptive prosocial norms by neighborhood and condition. Means and standard errors of responses are plotted in gray alongside model predictions (mean and 95% CI) (see Table 2, Model 2).

**Fig 4:**
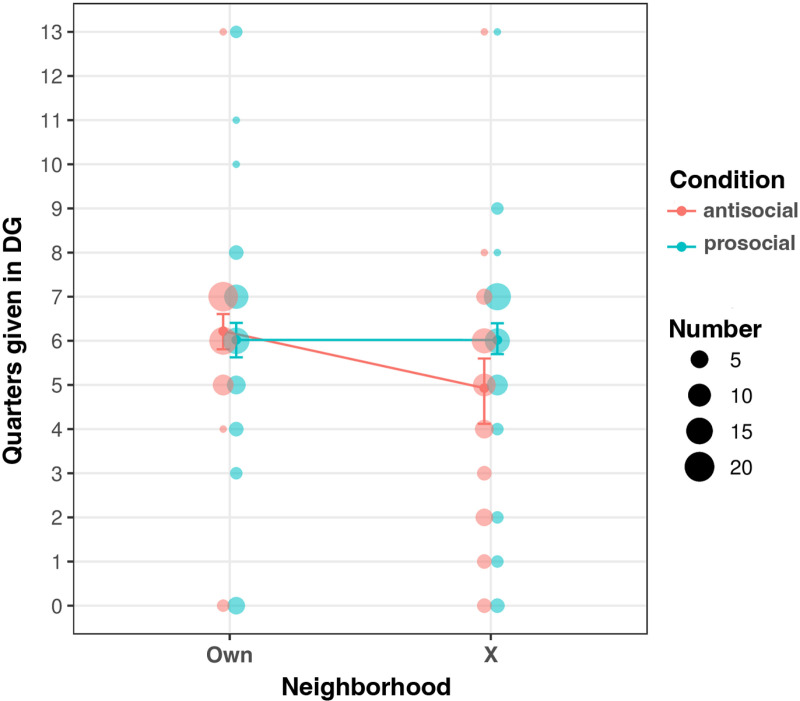
Quarters given in DGs by neighborhood and condition. Model predictions (mean and 95% CI; Model 2 in S2 Table) plotted alongside observations (99 participants); bubble size corresponds to number of children who gave away that number of quarters.

Overall, children characterized their Own Neighborhood as highly prosocial. Most of the children believed that more than half of their neighbors would behave positively and that few of their neighbors would behave negatively; they also perceived of adults in their Own Neighborhood as more prosocial than children (Fig 3 and Table 2). They exhibited highly prosocial behavior in the DG, with 72 of 99 participants giving away at least six of thirteen quarters, and 40 choosing to give away more than they kept for themselves (Fig 4 and Table S3).

### Do children infer different prosocial descriptive norms in Neighborhood X based on condition?

Descriptive prosocial norms in Neighborhood X diverged sharply dependent upon condition (Prosocial or Antisocial Neighborhood). Children assigned to the Antisocial condition believed that about half of the residents of Neighborhood X would behave negatively and a minority would behave positively (Fig 3 and Table 2). In contrast, those in the Prosocial condition viewed the residents of Neighborhood X as behaving more positively, and slightly less negatively, than the residents of their Own Neighborhood (Fig 3 and Table 2). The coinciding increased variation in descriptive prosocial norms in Neighborhood X, relative to Own Neighborhood, is revealed by comparison of the variance in slopes for the effect of Neighborhood X, conditioned on participant, to the variance in participants intercepts (Table 2).

### Do children extend their judgments of prosocial descriptive norms for Neighborhood X to unobserved behaviors?

The clear divergence of prosocial norms in Neighborhood X following condition remains when only those three behaviors in the norm questionnaire for which participants received no explicit information from the slideshow (i.e., recycling, helping an elderly neighbor carry groceries, and keeping a package that was supposed to be delivered to another household) are included in the analyses (S3 Fig and S1 Table). Thus, following their assigned condition, the children inferred divergent prosocial descriptive norms in Neighborhood X even for unobserved behaviors.

### Do children adjust their prosocial behavior in Neighborhood X based on condition?

Prosocial behavior in Neighborhood X deviated from that in Own Neighborhood for those children assigned to the Antisocial condition. Children in the Antisocial condition gave fewer quarters to a child in Neighborhood X (median = 5, Median Absolute Deviation = 1) than did children in the Prosocial condition (median = 6, MAD = 1), and they gave fewer quarters to a child in Neighborhood X than they gave to a child in their Own Neighborhood (median = 6, MAD = 1) (Fig 4). Considering DG behavior only within Neighborhood X, the odds that a child in the Antisocial condition gave fewer quarters than a child in the Prosocial condition are 242% higher (OR 3.42, 95% CI [1.64,7.15]; Model 2 from S2 Table fit to data from Neighborhood X only). For children assigned to the Prosocial condition, there is not a robust difference between the number of quarters given to a child in Neighborhood X compared to a child in their Own Neighborhood (Fig 4; OR for a child in the Prosocial condition giving away more quarters in Neighborhood X than in their Own Neighborhood is 0.93, 95% CI [0.39,2.25]; ordered logit model with varying intercepts for participants and covariates *gender* (boy) and *Neighborhood X*).

### Do children’s beliefs about prosocial descriptive norms within a neighborhood predict their DG allocations in that neighborhood?

We found a positive relationship between descriptive prosocial norms at baseline and the number of quarters given in the DG. That is, children who viewed their neighbors as more prosocial gave more quarters to another child in their Own Neighborhood. An increase of one standard deviation (SD) or more above the mean for descriptive norms is associated with a median of seven out of thirteen quarters (MAD = 1) given in the DG, compared with an overall median of six quarters (MAD = 1) in Own Neighborhood. Likewise, an increase of one SD in descriptive prosocial norms within Own Neighborhood is associated with a 49% increase in the odds of giving more quarters in Own Neighborhood (OR 1.49, 95% CI [1.03, 2.17] for ordered logit model with covariates of *gender* (boy) and *descriptive norms*). In other words, the odds are 1.49 times greater that a child who perceives of their neighborhood as highly prosocial (one SD above the mean) gave away more quarters in their Own Neighborhood compared to a child who described their neighborhood as average with respect to cooperation.

Similarly, children who viewed residents of Neighborhood X as more prosocial gave more quarters to a child in Neighborhood X. An increase of one SD in descriptive prosocial norms within Neighborhood X is associated with a 49% increase in the odds of giving more quarters to a child in Neighborhood X (OR 1.49, 95% CI [1.13, 1.96] for ordered logit model with covariates *gender* (boy) and *descriptive norms*).

When results from both Own Neighborhood and Neighborhood X are considered together, we see a strong influence of descriptive prosocial norms on prosocial behavior in the DG. Across both neighborhoods and conditions, for each one SD increase in descriptive prosocial norms, there is a 148% increase in the odds that a child gave away more quarters in the DG (OR 2.48, 95% CI [1.65,3.74]) (S2 Table, Model 1). This pattern persists, albeit with diminished strength (OR 1.88, 95% CI [1.01, 3.53]), when an interaction between the covariates *Antisocial condition* and *Neighborhood X* is added to the model (S2 Table, Model 3). At the same time, removing *descriptive norms* from the model that specifies an interaction between the covariates *Antisocial condition* and *Neighborhood X* decreases model fit (S2 Table, Model 2). These two observations suggest that perceived descriptive prosocial norms capture information relevant to DG behavior beyond that of assigned condition.

**Fig 5:**
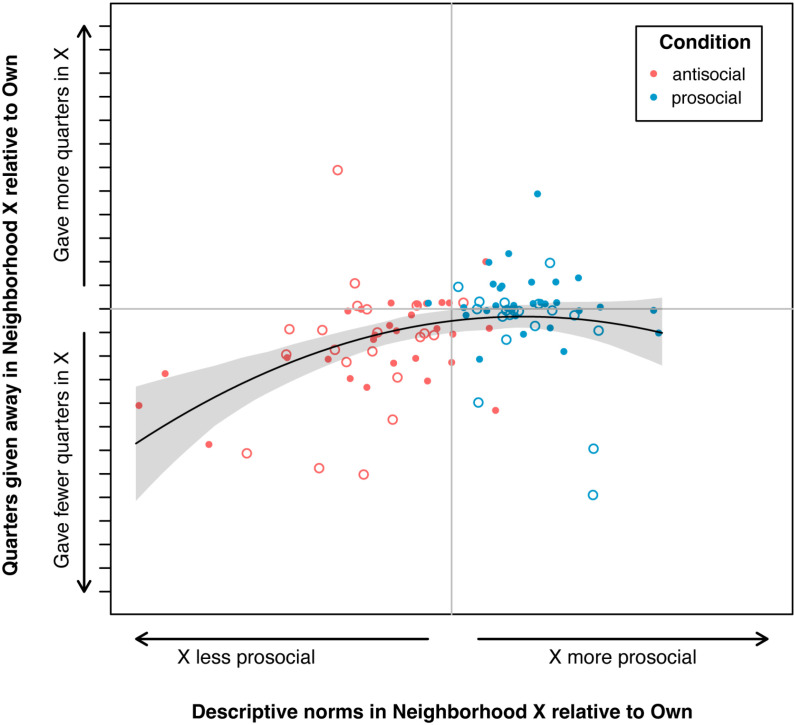
Relationship between change in descriptive norms (Own Neighborhood to Neighborhood X) and change in quarters given in DG. Open circles indicate participants who gave away over half of their endowment (seven or more quarters) in their Own Neighborhood; data from all participants represented. Tick marks on the y-axis represent a single quarter. Prediction line based on model with sole covariate being the quadratic change in norms; shaded area 95% CI.

In accordance with the observed relationship between descriptive norms and DG behavior, larger changes in descriptive norms are also associated with larger changes in the number of quarters given in the DG. That is, children who perceived a greater difference in prosocial behavior between their Own Neighborhood and Neighborhood X concomitantly altered their DG behavior more. The quadratic relationship between change in descriptive prosocial norms and change in the number of quarters given accounts for 17% of the variation in behavioral change (between Own Neighborhood and Neighborhood X) in the DG (R^2^ = 0.17, 95% CI [0.06, 0.38]; CI based on 1000 bootstrap replications) (Fig 5). This relationship persists when only the subset of norms for which children received no information in the slideshow are considered (R^2^ = 0.19, 95% CI [0.08, 0.40]; CI based on 1000 bootstrap replications).

### Is the change in prosocial behavior specific to Neighborhood X?

To address this question, we included the “Helping Task,” wherein the participants had the opportunity to help the researchers with a real-effort encryption task at the end of the experiment. Neither *Antisocial condition* nor *descriptive norms* for Neighborhood X are robust predictors of the number of encryption tasks completed. When these covariates are added separately to the baseline model resulting from stepwise model selection (baseline model includes *gender* (boy), *age*, and *helping task fun* (five-point scale)), the estimated coefficients are larger than the standard errors, and AIC increases (764 for each compared to 762). On the contrary, the addition of *descriptive norms* for Own Neighborhood improves model fit (AIC 744), and a one SD increase in descriptive prosocial norms in Own Neighborhood is associated with an expected 20% increase (95% CI [11%, 30%]) in the number of Helping Tasks completed (S1 Appendix). Similarly, *quarters given* in Own Neighborhood improves model fit (AIC 738) but *quarters given* in Neighborhood X does not. An additional quarter given in Own Neighborhood is associated with an 9% increase (95% CI [5%, 14%]) in the number of Helping Tasks completed. These results confirm that children’s prosocial behavior did not change outside of the context of Neighborhood X.

### Additional controls

#### Affect.

Children in the Antisocial condition were sadder after the slideshow according to the seven-point smiley/frowny face scale (median response of four (MAD = 1) than were children in the Prosocial condition (median response of three (MAD = 1)), with ordered logit regression analyses revealing a robust, positive effect of condition (*Antisocial condition* is associated with a 207% increase in the odds that child reported more negative affect (OR 3.07, 95% CI [1.46,6.46])). Sadder children gave away fewer quarters (OR 0.70, 95% CI [0.52,0.94]). However, once *descriptive norms* for Neighborhood X are included in the model, AIC decreases from 419 to 417 and *negative affect* is no longer a useful predictor of the number of quarters given (OR 0.81, 95% CI [0.58,1.13]), while *descriptive norms* still are. A one standard deviation increase in *descriptive norms* is still associated with a 52% increase in the odds a child gave away more quarters (OR 1.52, 95% CI [1.01,2.30]). Therefore, the difference in DG behavior in Neighborhood X between those assigned to the Antisocial condition, versus those assigned to the Prosocial condition, cannot be attributed solely to an increase in negative affect.

#### Injunctive norms.

Participants expressed highly prosocial personal injunctive norms (S3 Table). This did not change after the slideshow of Neighborhood X; inclusion of the covariates *Neighborhood X* and *Antisocial condition* do not improve model fit, and inspection of the coefficients for the fitted model specifying an interaction between *Neighborhood X* and *Antisocial condition* shows that the covariates are not particularly informative about responses to personal injunctive norms survey items.

## Discussion

The children in our study demonstrated that they could infer the prosocial norms of a new neighborhood, including unobserved norms, and adapt their behavior to align with the norms of the new neighborhood. Our analyses suggest that this behavioral modification was precipitated by the recognition of different descriptive norms in operation in Neighborhood X. More specifically, our results support our hypotheses that, by middle childhood, children have the propensity to 1) rapidly infer local descriptive pro- and antisocial norms in a novel social environment, 2) extend these inferences to unobserved behaviors, and 3) adjust their behavior in accordance with those norms 4) without altering their behavior outside of the novel social environment.

That is, children in the Prosocial condition described Neighborhood X as far more prosocial than did children in the Antisocial condition. The effect of condition on descriptive norms in Neighborhood X remained when only those norm questionnaire items for which there was no correlate in the slideshow were analyzed. Similarly, although the participants did not receive information about DG or other sharing or giving behavior in Neighborhood X, those in the Prosocial condition gave more in a DG with another child in Neighborhood X than did those in the Antisocial condition (DG behavior in the Prosocial Neighborhood X was similar to that in Own Neighborhood). Indeed, descriptive prosocial norms predicted DG behavior in both Own Neighborhood and Neighborhood X, and the change in descriptive prosocial norms between Own Neighborhood and Neighborhood X accounted for 17% of the variation in change in the number of quarters given. Thus, the participants were not merely imitating what they observed; rather, it appears that the children extracted a general understanding of the descriptive prosocial norms operating within Neighborhood X and extended those norms to unobserved behaviors or situations, akin to the cross-norm effect described by Keizer and colleagues [26,28]. Importantly, assigned condition and perceived descriptive norms for Neighborhood X did not predict behavior in a separate Helping Task, suggesting that observed changes in DG behavior between Own Neighborhood and Neighborhood X were specific to Neighborhood X. Taken together, our results indicate that key components of human norm psychology, including the rapid inference and adoption of prosocial norms in a novel milieu, are in operation by middle childhood.

While our main interest was in evaluating a change in perceived norms and prosocial behavior in Neighborhood X relative to Own Neighborhood, our finding that descriptive prosocial norms in Own Neighborhood predicted DG behavior in Own Neighborhood—as well as behavior in the Helping Task—is also of note. These results are consistent with studies that have shown that, by middle childhood, children’s prosocial behavior in experimental games is influenced by norms of game play [44–48] and reflects the choices local adults make [7,44], as well as research that has demonstrated an effect of local norms on prosocial and antisocial behavior in adults [15,20–22,24,26–28]. However, to our knowledge, this is the first study to show that variation in prosocial behavior in children is, at least in part, explained by variation in perceived descriptive prosocial norms in the children’s own milieux.

One shortcoming of our study is the lack of diversity among participants, which extends to their Own Neighborhoods. The children who participated in the study were predominantly white, from stable, bi-parental homes (the majority of which were owned by their parents, suggesting high SES) and, following their own assessments, highly prosocial neighborhoods. They also behaved prosocially in the DG, with approximately 72% giving away six or more of the thirteen allocated quarters in their Own Neighborhoods. Thus, there was little possibility for those in the Prosocial Neighborhood X to increase their prosocial behavior without giving away considerably more than half their endowment to the DG recipient, which would have conflicted with a strong preference for equitable distributions that is present even in young children [41,66–68] in so-called WEIRD (western, educated, industrialized, rich, democratic) populations [69]. One way to address this shortcoming would be to increase the DG choice set, as Bonan et al. [43] did in a series of DGs conducted with children in El Salvador. When the dictator had the opportunity to either give to or take from the recipient (who received an endowment half the size of the dictator’s), children were less likely to give anything at all, and gave less when they did, compared to in a traditional DG. Were we to repeat our experiment in the same population with a similar choice set, we might expect a larger discrepancy in recipient outcomes between conditions in Neighborhood X; i.e., we would expect participants in the Antisocial condition to take more than they did in their Own Neighborhood and those in the Prosocial condition to potentially give more than in Own Neighborhood (note that there is not a marked difference in prosocial norms between Own Neighborhood and the Prosocial Neighborhood X).

The lack of diversity in our sample also leads us to question whether the behavioral plasticity we observed, with children in the Antisocial Neighborhood X giving fewer quarters in the DG, would extend to a sample of children who were introduced to a Prosocial Neighborhood X that was considerably more prosocial than their Own Neighborhoods. Studies conducted with children in diverse countries and non-industrialized cultures have shown that equitable distributions in the DG, as seen in Own Neighborhood in this study, cannot be considered the norm [10,44]. As noted above, while some studies find an inverse relationship between SES and prosocial behavior, others have linked lower SES to less prosocial behavior in children [10,41,66,70], potentially because disadvantage and stress can have negative effects on two components of cooperation, i.e., trust and the propensity to forgo smaller, more immediate rewards in favor of larger, distant rewards [71,72]. Thus, it may be difficult to select participants who perceive of their Own Neighborhoods as less prosocial without varying SES at the same time; correspondingly, antisocial behaviors in Neighborhood X (such as graffiti) might inadvertently signal SES as well (see, for example [73]).

Putting the confounding factor of SES aside for a moment, there is also reason to question whether children would adjust their behavior to conform to norms that are more prosocial than those in their own neighborhood as readily as they would adjust their behavior to be more antisocial. On the one hand, a demonstrated bias towards that which is negative is observed across a host of domains [74,75]. Specifically, experiments have demonstrated that children are more likely to engage in negative direct or indirect reciprocity [35,36,76] than positive reciprocity and that antisocial modeling has a greater influence on behavior than prosocial [30]. On the other hand, there is also evidence that prosocial modeling has at least as great an influence on behavior as antisocial [31,46,47], suggesting that there are likely complex interactions with age and culture [30]. Additionally, using a series of economic games, Westhoff and colleagues [77], demonstrated that while children will adjust their behavior to be less prosocial in less prosocial environments, only teenagers and adults also readily alter their behavior to be more prosocial in prosocial environments, a result driven by an increased tolerance of disadvantageous inequity. Thus, it may be that, ontogenetically, children first adjust their behavior to align with antisocial norms and only later adjust them to align with prosocial norms. Extension of the experiment to a sample of children representing a wider age range would be informative.

Consideration of the above-mentioned, complex relationship between prosocial behavior and SES leads us to question whether children in the study considered the residents of Neighborhood X and, critically, the child with whom they played a DG in Neighborhood X, as part of an out group. Even at a young age, children demonstrate an in-group bias in resource allocation tasks [67,78], and norm manipulation studies have demonstrated that the extent to which a subject identifies with the reference group for a particular prosocial norm is of consequence [20,50]. While the current study was explicit as to whom the reference group for the norm manipulation was (i.e., the neighborhood), groups may be construed in many ways, and, given the prosocial backgrounds of the children in the current study, those in the Prosocial condition might have identified with residents of Neighborhood X on the basis of their similarly prosocial behavior—i.e., a norm-based group membership—while those in the Antisocial condition might not have. Thus, it is possible that children in the Antisocial condition were at least in part motivated to give fewer quarters to children in Neighborhood X because they considered them members of an out-group.

Another possible interpretation of the DG results—which may hinge upon whether participants considered the DG recipient in Neighborhood X an in- or out-group member—is that participants in the Antisocial condition gave less in the DG in Neighborhood X because they wished to punish the recipient (or, on the flip side, reward the Prosocial Neighborhood X DG recipient). There is strong experimental evidence that adults will pay to enforce prosocial norms and that such action is effective at sustaining higher levels of cooperation [79,80]. A variant of the DG, in which a third party has the option to reduce the payoff to the dictator at a cost to herself (Third-Party Punishment Game, 3PPG) [81], has been widely used to demonstrate that adults will pay a cost to enforce prosocial norms, although this varies culturally [82]. A majority of children in industrialized populations also engage in such “altruistic punishment” by around six to eight years of age [3,83]. Thus, it is possible that participants expected the anonymous DG recipient in the Antisocial Neighborhood X to be antisocial (a participant’s perception of a given group’s descriptive norm for a given behavior should be indissolubly linked to the participant’s expectation of a random group member’s behavior) and desired to punish them by giving them fewer quarters than a child in their Own Neighborhood. (This behavior would not be considered *costly* or *altruistic* punishment, as the payoff to the dictator in a traditional DG only increases by withholding quarters from the recipient.)

However, there are a few arguments against this explanation for DG behavior in the Antisocial Neighborhood X. Punishment of the dictator in the 3PPG varies depending on the group membership of the three players in the 3PPG, as reviewed by McAuliffe and Dunham [84]. Multiple studies show that the “third party” is less likely to punish the dictator for stingy behavior when both the dictator and recipient belong to a different group than the third party [81,85,86]. Thus, we might expect participants in the Antisocial Neighborhood X DG to be less motivated to punish antisocial behavior if they perceived both the victims and perpetrators of the behavior to be members of an out-group. At the same time, central to Broken Windows Theory [87] is the idea that antisocial behavior is perceived as evidence that “nobody cares”—i.e., evidence of a lack of prosocial norm enforcement, thus encouraging more antisocial behavior (rather than norm enforcement). In support of this theory, a multi-neighborhood study in Chicago found that collective action (informal social control and social cohesion) mitigated the positive relationship between disadvantage/residential instability and interpersonal violence [73]. Evidence that antisocial descriptive norms are a negative predictor of punishment also comes from two “lab in the field” studies. Participants who assessed their neighbors as likely to behave antisocially (e.g., cheat on public transport fare) were less likely to expect their neighbors to punish them, and more likely to take from their neighbors, in a 3PPG; a subsequent norm manipulation, in which participants were informed that their neighbors behaved prosocially, resulted in an increased expectation of punishment [27].

While we cannot rule out that a punitive sentiment was instrumental in the observed DG behavioral shift, we note that anger or a desire to punish a member of the Antisocial Neighborhood X—and, conversely, positive sentiments and a desire to reward a member of the Prosocial Neighborhood X—are not mutually exclusive with a desire to align one’s behavior to recognized norms but may be an important motivator; the role of emotions in norm conformity remains under-explored [88,89]. Indeed, Fehr and Gächter [80] found that anger motivated the punishment of defectors in a Public Goods Game, allowing groups to achieve and sustain higher levels of cooperation.

Clearly, much could be learned by extension of the current study to a more diverse sample population and more expansive DG choice set as well as a wider range of ages. At the same time, in order to elucidate the cognitive and motivational mechanisms at play in any observed behavioral shifts, future work should incorporate questions about punitive or positive sentiment towards the DG recipient and the extent to which participants identify with or trust residents of Neighborhood X.

## Conclusion

Using a novel methodology, we found that by middle childhood, children have the propensity to 1) rapidly infer the local pro- and antisocial descriptive norms of a new social environment, 2) extend these inferences to norms for unobserved behaviors, and 3) apply the inferred norms when engaging in a social dilemma—4) but only in that specific social environment. Our results are consistent with the norm psychology hypothesis, which proposes that humans possess evolved cognitive and motivational mechanisms for the acquisition, application, and enforcement of local norms [14]. Furthermore, they suggest that the concept of a stable “cooperative phenotype” [90,91] may need to be refined, or at least further explored in varying social environments. Of theoretical importance, norm psychology could, in concert with transmission biases [14], facilitate the maintenance of cultural differences in prosocial behavior despite migration, which would enable the cultural group selection of prosociality [92–94].

## Supporting information

S1 Appendix Stimuli, data cleaning, and validation. (PDF)

S2 Appendix R code used to analyze data. (TXT)

S1 Dataset Data collected and analyzed as described in Materials and methods section and stored in.csv file. (CSV)

S1 Key S1 Dataset variable key. Description of variables in dataset, stored in.csv file. (CSV)

S1 Fig Influence of parental desire for prosocial behavior (PDI) on DG contributions. Means and standard errors of number of quarters given are plotted in gray alongside model predictions (mean and 95% CI). The model specifies an interaction among *PDI*, *Antisocial condition*, and *Neighborhood X* (S1 Appendix). High and low PDI represent a median split.
(TIF)

S2 Fig Adult presence in room and number of correct/incorrect child responses. Counts of outcomes for each of 13 questions for which the response could be assigned a “correct/incorrect” value. Missing responses were treated as incorrect. As ordered, the 13 questions/instructions are: child’s age; child resides in multiple neighborhoods; successful use of drag and drop feature at first attempt during tutorial; number of balloons observed in box in during drag and drop tutorial; number of quarters in quarter game, Own Neighborhood (ON); identification (self/other) of recipient of quarters dragged to “Other Child’s Box” (ON); other child in quarter game lives in ON (true/false) (ON); quarters can be used to get digital prizes at end of study (true/false) (ON); successful clicking on target at first attempt during HeatMap tutorial; number of quarters in quarter game in Neighborhood X (NX); identification (self/other) of recipient of quarters dragged to “Other Child’s Box” (NX); other child in quarter game lives in NX (true/false) (NX); quarters can be used to get digital prizes at end of study (true/false) (NX).
(TIF)

S3 Fig Descriptive prosocial norms by neighborhood and condition; subset of norms for which children received no information in slideshow. Means and standard errors of responses are plotted in gray alongside model predictions (mean and 95% CI) (see S1 Table, Model 1).
(TIF)

S1 Table Descriptive prosocial norms modeled as dependent upon an interaction between condition and neighborhood for a subset of the norms survey data. Ordered logit regression. Dataset includes only those questions for which there was no information in the slideshow. Predictions from Model 1 depicted in S3 Fig. Parentheses contain 95% confidence intervals for ORs. Standard deviations given for estimated variance components. “Correlation” refers to correlation between estimated slopes, intercepts.
(PDF)

S2 Table Number of quarters given in DGs dependent upon descriptive prosocial norms and condition. Multilevel ordered logit regression. Parentheses contain either standard errors (variance components) or 95% confidence intervals (OR). Predictions from Model 2 plotted in Fig 4.
(PDF)

S3 Table Personal injunctive prosocial norms dependent upon condition and neighborhood. Multilevel binomial logistic regression. Parentheses contain either standard errors (variance components) or 95% confidence intervals (OR).
(PDF)
