# Supplementary material for: Children adjust behavior in novel social environment to reflect local prosocial norms inferred from brief exposure
Source: PLoS One. 2025 Jul 9;20(7):e0325984. doi: 10.1371/journal.pone.0325984 (PMC12240362; doi:10.1371/journal.pone.0325984)
Supplement: S1 Appendix — (PDF) [file pone.0325984.s001.pdf]

S1 Appendix: Stimuli, data cleaning, and validation

Children adjust behavior in novel social environments to reflect local prosocial norms inferred from brief exposure

Kari Britt Schroeder[1*,#a], Laura Nelson Darling[1,#b], Peter R. Blake[1]

[1]Department of Psychological and Brain Sciences, Boston University, Boston, MA, USA

[#a]Current address: Independent researcher, Nyon, Switzerland

[#b]Current address: Boston Child Study Center, Boston, MA, USA

* Corresponding author

Email: kari.britt.schroeder@gmail.com (KBS)

Neighborhood X Stimuli

Creation

With the help of volunteers and research assistants associated with the Social Development and Learning Lab (SDLL), we photographed all stimuli in two neighborhoods within Boston and Cambridge (Fig 2). The use of fabricated images of behavior to introduce Neighborhood X enabled us to retain as much ecological validity as possible while also controlling for confounding variables that are often correlated with cooperative norms, such as poverty [1]. We selected cooperative norm situations (Table 2) which satisfied the following conditions: 1) representation with one or two photos, 2) no physical harm, and 3) symmetry of people and setting for both conditions. We endeavored to include situations involving children and adults as well as situations involving consequences for both a single individual and the public good.

Validation

Twenty-four children (15 male) ages eight to 11 years, inclusive, were recruited at Boston Common, a large public park in downtown Boston, and at the Social Development and Learning Lab at Boston University. Participant age was chosen based on the ages we expected to focus on for the main (online) study, but we included 8-year-olds as well to increase the participant pool, as visitors to Boston Common are predominantly younger children. Research assistants approached families who were visiting the park accompanied by children that appeared to be in the appropriate age range. The families were asked to participate in a 15-20 minute study in the park that re-

quired their children to look at and rate (with respect to acceptability and frequency of behaviors) pictures of people either violating or upholding cooperative norms. Participants picked out a small toy valued at $1.50-$3.00 as a thank-you gift.

The acceptability of each behavior was assessed by asking subjects “What do you think about this behavior?” Answers were constrained to a five-point scale, anchored at “Very bad” and “Very good.” The frequency of each behavior was assessed by asking subjects “Do you think many people would do this?” Answers were constrained to a five-point scale, anchored at “No one would” and “Everyone would.” Data on the frequency of the behaviors is limited to 22 subjects as two children responded only “yes” or “no” when asked “Do you think many people would do this?”

Prompted by qualitative assessment of subject responses, we changed the text for two of the eleven norm stimuli to further clarify the prosocial nature of the depicted behavior. We did this while the stimuli validation study was ongoing. We altered the Prosocial condition graffiti situation, which depicts a women painting over graffiti on a wall, so that it reads “We saw this woman cleaning up graffiti on a wall at the park” rather than “We saw this woman painting over graffiti on a wall at the park.” The Prosocial condition gum situation, which depicts a boy throwing his gum away (rather than sticking it on a bench), was changed from “We saw this boy take his gum out of his mouth and throw it away” to “We saw this boy take his gum out of his mouth and

throw it away in a trash can." We have data on the acceptability and frequency of the prosocial situation with altered text for eleven and ten subjects, respectively.

To confirm that the participants assigned prosocial situations a positive rating and antisocial situations a negative rating, we fit binomial generalized linear models to the data. For each prosocial situation, ratings were categorized as one (good, very good) or zero (okay, bad, very bad), and for each antisocial situation, ratings were categorized as one (bad, very bad) or zero (okay, good, very good). Because of quasi-complete separation for some of the situations, we fit the models with the bias-reduction method of [2], as implemented in the R [3] package brglm [4]. Data plotting and model fitting were conducted with the R packages ggplot2 [5]and rethinking [6,7], respectively.

Visualization of the data revealed that the ratings were strongly patterned according to whether the situations were prosocial or antisocial, with prosocial situations primarily rated as good/very good and antisocial situations primarily rated as bad/very bad. This pattern is even starker when only the ratings assessed subsequent to minor editing of the text for two situations, *graffiti* and *chewed gum*, as described above, are considered; this suggests that the revisions we made to these stimuli were appropriate. Formal analysis, via fitting of binomial generalized linear models, confirmed that the prosocial framing had a robust positive effect on the log odds that the situation was rated as good/very good (i.e., the estimated logit coefficients and 95% CI for all prosocial stimuli were positive), and the antisocial framing had a robust positive effect on the log

odds that the situation was rated as bad/very bad (i.e., the estimated logit coefficients and 95% CI for all antisocial stimuli were negative). Neither the age nor the sex of the participant appears to have affected the given rating.

Present study

Participant recruitment and participation

We employed the following exclusionary criteria during recruiting: 1) lack of home access to a desktop/laptop computer and the internet (the study was conducted online via the Qualtrics software platform), 2) diagnosis of dyslexia, 3) diagnosis of autism, and 4) psychoactive drugs (this criterion because of a potential follow-up genetic study). We further limited participation to children whose biological parents identified as being primarily of European ancestry because the actors in the stimuli photographs would be likely be viewed as white, and we wanted to maximize the likelihood that participants would make inferences based upon the behavior of the actors rather than focusing on in-group or out-group membership based on race or ethnicity.

One hundred and five children between the ages of 9 and 11 years, inclusive, participated in the online session over a period of eight months. Sessions were completed, on average, 18.2 days after either the mother (98) or father (seven) completed the online questionnaire (five parent surveys were completed subsequent to the child session). For unknown reasons, four recruited children never participated in the online session although their parents completed the questionnaire.

Child participants largely met the recruitment criteria, as indicated by their parents in the online questionnaire. One hundred of the parents confirmed that both biological parents were of European ancestry, and all parents confirmed that their children had not been diagnosed with autism or dyslexia. Three parents reported that their children were taking psychoactive drugs (one of these children is included in the final dataset).

One hundred and one of the children completed the full session. The four children who did not complete the entire session either stopped for unknown reasons (two) or encountered a technical problem with a webpage (two). All four of these children completed the pre-stimuli questions (this included a questionnaire about generalized trust, the data for which were not included in the current paper), and one also completed the first (Own Neighborhood) DG. Only one of these children was exposed to the stimuli (Prosocial condition), so for three of the four incomplete sessions, failure to complete the session cannot be attributed to the stimuli. One of the two children who encountered a problem with a webpage attempted to complete the session on two separate occasions but encountered the same problem both times.

Data cleaning

Exclusion of participants, interrupted sessions, and problems with interface

Of the 101 children who completed the entire session, two are excluded from the final dataset. One child started the session but had to leave for school immediately after viewing the stimuli.

We provided an abbreviated version of the session for him, which he commenced over seven hours later, but he viewed the stimuli twice and play the two DGs at different times. Thus we excluded data from his sessions. The other child completed the full session without obstacle; however, there was a discrepancy in the number of quarters he indicated should be given to the other child in the first DG (i.e., his text response did not match his drag and drop response).

Of the final dataset, there are four participants for whom further comment is required. Two children started the survey on a smartphone or tablet. These initial attempts were abandoned due to technical difficulties and the children both restarted the session on a laptop or desktop computer (one started over within minutes, and the other started over on the subsequent day). Neither of these two children viewed the stimuli in their initial attempts, and thus while their initial attempts are not included in the final dataset, their subsequent complete sessions are.

Two different children were unable to successfully use a drag and drop interface in both the trial prior to the DGs and in the DGs; one of these children was also unable to successfully click on an object in both the trial and the stimuli set. It is possible that the children were frustrated by the experience, and this frustration could affect their responses; however, potential frustration should be captured by our affect measure. Data from both of these children are retained in the final dataset as they were able to use the text boxes to allocate quarters in the DGs.

Final dataset

Thus, the final dataset includes 99 children. There are data for 49 participants in the Antisocial condition, 25 of which are girls, and 50 participants in the Prosocial condition, 25 of which are girls. There are data for nine or ten participants of each sex, in each condition, for all ages except the following: 11-year-olds (five or six participants for each condition, both sexes), 9-year-old boys in the Antisocial condition (five participants), and 10-year-old boys in the Antisocial condition (13 participants).

The final dataset includes three sibling pairs, one of which is a pair of fraternal twins. All three sibling pairs were assigned to opposite conditions, and the parents were instructed to keep the siblings separate and not let them communicate until both had completed the study; two pairs completed the session simultaneously, and the siblings in the third pair completed it consecutively, without a time lag.

Missing data

For the final dataset, very few questions were left unanswered. For questions about descriptive and injunctive norms in Own Neighborhood, 0.5% and 0% of questions, respectively, were left unanswered. For questions about descriptive and injunctive norms in Neighborhood X, 1.6% and 0.4% of questions, respectively, were left unanswered. Note that participants were unable to proceed to the next page without completing the text box portion of each DG.

Participant experience

Children took, on average, 46 minutes and 56 seconds (σ = 31 minutes, 28 seconds) to complete the session, including prize selection and viewing of the debriefing video (approximately 5 minutes, 30 seconds). The shortest session was completed in 20 minutes and 27 seconds. Session length was recorded as greater than one hour for 13 children. Of these 13 participants, eight left the browser open on either the prize selection or debriefing video webpage for over ten minutes (two participants never navigated past these final webpages, resulting in sessions automatically shut down by Qualtrics after four hours). Excluding these eight participants, the longest session was 95 minutes, 5 seconds.

Approximately one-fifth of the participants (21.2%) said another person was in same room at start of study (2.0% of these participants indicated the other person was an adult). Only three of these indicated that the person was talking with them about the study. We investigated whether the presence of another person in the room influenced the children's responses; see *External influences on child responses* below.

Children were shown a sampling of potential digital prizes at the start of the session and asked how much they liked these prizes; they were also told that the final selection would depend on their choices during the study as well as the digital devices to which they have access (this question was posed on a subsequent webpage and not during viewing of the sample prizes). About half of the children (50.5%) indicated they saw prizes they liked, and only 8.3% did not see any

they liked, with the rest unsure.

At the end of the session, children selected, via drag and drop, images of their top three choices for prizes. Every child received at least one, if not two, of their top three choices for prizes. We gave children as many of their top choices as possible as long as the total amount was within approximately one dollar of the child's total earnings from the session. Prize choice was strongly patterned, with a few prizes extremely popular, which suggests that we offered prizes that many of the participants liked. The most popular items were the games (apps) Minecraft (17) and Plants Vs Zombies (17). A subset of the books on offer was very popular as well: *Ungifted* by Gordon Korman (12), books from the Percy Jackson and Heroes of Olympus series by Rick Riordan (11), and *Boy* by Roald Dahl (7). MP3s were relatively unpopular.

Validation of responses

No researcher was present to guide the child through the study; thus, we assessed participant compliance and engagement with the following measures: time spent on the study, completion of study components, comprehension checks following instructions for the DGs, and assessment of Qualtrics Heat Map data. Moreover, we searched for external influences on child responses to questions and instructions for which there was a single correct response and on child behavior in the DGs (S1 Fig and S2 Fig).

Engagement with stimuli

Two measures enable an assessment of how engaged participants were with the stimuli (exposure to Neighborhood X): 1) Heat Map data collected with Qualtrics, which shows whether/where upon the image(s) the participant clicked, and 2) time spent viewing the stimuli.

For each stimulus, subjects were instructed to “Click on the part of the picture you think is most important.” Of the 99 participants in the full dataset, 91 clicked on all stimuli at least one time. One individual never clicked on any of the stimuli (as mentioned above, the same individual failed to use the drag and drop interface), and another individual failed to click on the majority of stimuli (seven of eleven stimuli). The remaining six individuals who did not click on all stimuli only failed to click on one stimulus (five individuals) or two stimuli. Thus, almost all of participants interacted with almost all of the stimuli as directed.

To assess whether the participants were attending to the aspects of the stimuli relevant to social behavior, we randomly selected nine participants for each condition and visually reviewed where upon each image they clicked. For the Antisocial stimuli, only one of 99 assessed clicks was not on the actor, the human recipient of action, or one of the objects (e.g. candy, mail, litter or trash can) mentioned in the text (the child clicked on the fire hydrant in the dropped mail stimulus). Similarly, for the Prosocial stimuli, only one of 99 assessed clicks was not on the actor, recipient of action, or one of the objects mentioned in the text (the child clicked on a grassy area next to dog poop in the dog poop stimulus).

Thus, it appears that the children chose as "most important" those aspects of the stimuli which we also considered relevant. Because of a lack of variation in whether the participant clicked on a relevant aspect of the stimuli, we did not consider this variable in downstream analyses.

The median time children spent viewing all 11 stimuli is 2 minutes, 44 seconds (MAD = 60 seconds). Median time spent viewing the stimuli is 39 seconds longer for participants who viewed the antisocial stimuli (2 minutes, 51 seconds; MAD = 34 seconds) than those who viewed the prosocial stimuli (2 minutes, 12 seconds; MAD = 34 seconds). Negative binomial regression analysis confirms that children in the Antisocial condition spent 17.3% (95% CI [3.0%, 33.7%]) longer viewing the stimuli than those in the Prosocial condition and that older children spent less time on the stimuli (11.8% less time per year; 95% CI [19.4%, 3.6%]). The total number of words in the stimuli for the Antisocial Neighborhood X is 163, as opposed to 174 for the stimuli used for the Prosocial Neighborhood X, so it is unlikely that the longer time children in the Antisocial condition spent viewing the stimuli was due to greater reading time. A robust association between sex and time spent viewing stimuli was not observed.

Computer competence and comprehension of the Dictator Game

Prior to viewing the stimuli, participants were given a brief tutorial on using test boxes and radio buttons, and all participants then successfully used text boxes and radio buttons.

Participants were also given a brief tutorial on clicking on a target on the screen. Following the

tutorial, 91 of the 99 participants in the final dataset correctly clicked on a trial target, two did not click on anything (one of these, as mentioned above, never successfully used the drag and drop interface either), and six clicked off-target.

For both DGs, participants were instructed to indicate the number of quarters to be allocated to themselves and the other child via both text boxes and drag and drop of images of quarters. Prior to the first DG, participants were given a brief tutorial of the drag and drop interface, followed by a trial. Three participants failed to successfully complete the trial drag and drop (participants were given only one try). Of these, one subsequently successfully allocated quarters via drag and drop but, as described above, two failed to use the drag and drop interface and indicated quarter allotments only via text boxes.

Prior to both DGs, participants were given instructions for the game, as follows:

Now you will play a game! You will play this game with another Child in Your Neighborhood (Neighborhood X). There are 13 quarters in the game. You have to decide where to put all 13 quarters. There are two boxes you can put the quarters into. One box is Your Box. The second box is the Other Child's Box.

All of the quarters that you put in Your Box will be yours. You can use these quarters to get one or more songs, books, or games at the end of the study.

All of the quarters that you put in the Other Child's Box will go to a Child in Your Neighborhood (a Child in Neighborhood X). For each quarter you put in the Other Child's Box, this child will get a real quarter.

This other child will never know your name. You will never know who the other child is, either.

They were also asked a series of questions to assess their understanding of the game. Each time, one child (a different child each time) indicated the wrong number of quarters in the game (13 is the correct answer) via multiple choice. Prior to the first DG, 93 participants correctly answered on the first try, via multiple choice, that the quarters put in the Other Child's Box would go to Another Child, not to themselves. Of the six participants who did not respond correctly, three were asked again (this check was not implemented until approximately five weeks after commencement of the study), and two of these three answered correctly on the second try. Participants who answered this question correctly on the first or second try (95) were then asked if the child lived in his/her neighborhood; of these, 11 answered incorrectly and one did not answer.

Prior to the second DG, 95 of the 99 participants correctly answered, on the first try, via multiple choice, that the quarters put in Other Child's Box would go to Another Child, not to themselves. Of the four who did not respond correctly, one was asked again (see above), and this child answered correctly on the second try. Participants who answered this question correctly were then asked if the child lived in Neighborhood X; of these, 13 answered incorrectly, and one child did not answer. Of concern is whether children responded “No” to this question, or the corresponding Own Neighborhood question, because of disbelief, rather than error or lack of understanding of instructions. If this were true, we would expect older children to be less gullible. However, there is a lack of evidence from multilevel logistic regression that the child's age, sex, the condition to which she was assigned, or the neighborhood to which the question referred (i.e., Own Neighborhood or Neighborhood X) affected whether this question was answered correctly (for

all fixed effects coefficients--age, sex, condition, neighborhood--the estimated standard errors were substantially larger than the mean estimates).

Additionally, the webpages for both DGs, which the participants viewed subsequent to answering the DG comprehension questions, contained two additional textual indicators of the other child's neighborhood (either Own Neighborhood or Neighborhood X). One indicator was in the instructions above the quarter drag and drop interface: “Check to be sure you know… which box is for the Other Child in Your Neighborhood” (or “the Child in Neighborhood X” for the second DG). The other indicator was placed below the drag and drop interface, next to the text box in which participants were to enter how many quarters to allocate to another child. Participants were prompted to indicate how many quarters were allocated to “the Other Child in Your Neighborhood” (or “the Child in Neighborhood X”).

Two children incorrectly answered the question of whether the quarters put in the Other Child's Box would go to Another Child prior to both DGs. Both of these children took the study before a change was implemented wherein the question would be posed again if participants answered incorrectly. However, both answered all other test questions correctly for both DGs, and moreover, both demonstrated consistency in the number of quarters allocated to themselves and another child via drag and drop and via text boxes, so we infer that these children understood which amount was being allocated to themselves and which amount was being allocated to another child.

Prior to both DGs, 3.0% of children incorrectly indicated, via multiple choice, that they could not use the quarters in the game to get one or more digital prizes.

Overall, the remarkably high consistency between drag and drop and text responses for both DGs (there was one inconsistency, as mentioned above, out of 194 potential inconsistencies; this child answered all other test questions correctly but was excluded from the final dataset) suggests general comprehension of the quarter distribution schema. The high consistency between drag and drop and text responses also suggests that the participants were carefully attending to their actions in these tasks.

Triangulation of child age

As a separate check of child engagement with the study, computer comprehension, and accurate merging of data from the child sessions and parent surveys, we checked for consistency in child age from three different sources. At the start of the study, children entered their age in a text box to assess their familiarity with the use of text boxes. This value was checked against the child's age as calculated from our records of the child's date of birth and the date the child participated in the online session. The child's data of birth according to our records (database of children recruited to the SDLL) were then checked against the birth month and birth year as entered by the parent.

Three child participants in the full dataset gave incorrect ages; one of these entered an age that was wildly incorrect (2 years) and likely a typo, and the other two entered ages that were within five months of their true age, which thus may represent cognitive mistakes or wishful thinking. A fourth inconsistency was found that is likely due to error in our records, as the parent and child gave concordant information that differed from what we had on record.

External influence on child responses

Because we did not observe the child's participation in this study, we conducted a series of analyses to search for an effect of external influences on child responses. We assessed 1) whether parental desire for prosocial behavior in children is predictive of DG giving, and 2) whether the presence of another person in the room during the study is predictive of correct responses to questions or of 3) DG giving.

Parental desire for child prosociality

As mentioned in the **Materials and methods** section of the main text, parents were asked to complete a survey prior to the participation of their child. With respect to child-rearing, we asked parents, "In your opinion, how important is it for children to develop or learn the following?" Parents responded via a seven-item scale, with one corresponding to "Not at all important" and seven corresponding to "Extremely important." Three of the nine items on this questionnaire are pertinent to prosociality: "Learn to help others," "Learn to care for the well-being of others," and "Learn to cheer up others."

We created a “parental desire for child prosociality index” (PDI), using an approach similar to the descriptive norm indices. That is, we treated these three questions as repeated measures of parental desire for child prosociality and analyzed the data using binomial logistic regression models (given the paucity of variation in responses, we collapsed responses across the lower six bins of the scale) in R [3] with the packages glmer2stan [8], Rstan [9], and rethinking [6,7], with varying intercepts for individual parents and a fixed effect for *boy* as well as for two of the three questions. Each parent’s score for the PDI is the mean estimate of the parent's intercept.

We then assessed a potential effect of PDI on behavior in the DG, using multilevel ordered logit regression, starting with the base model previously used to asses the affect of condition and norms on DG behavior (S3 Table, Model 2). Model fit, as assessed with the deviance information criterion (DIC), improved when a three-way interaction among *PDI*, *Antisocial condition*, and *Neighborhood X* was added (DIC of 607 for the base model compared to DIC of 603). This effect of PDI could result from parental teaching, shared environment, shared genes, or real-time parental influence in the study. Our concern is whether there was real-time parental influence in the study; the plotted predictions suggest that this was not the case, because the effect of condition on DG behavior is still clearly present when PDI is considered; indeed, there is a greater (negative) change in DG behavior between Own Neighborhood and Neighborhood X for those children whose parents’ score for the PDI was above the median, perhaps indicative of greater disillusionment (S1 Fig).

When *descriptive norms* are added to the model with a three-way interaction among *PDI*, *Anti-social condition*, and *Neighborhood X*, the estimated OR for *descriptive norms* decreases only slightly (DIC 601) (OR 1.73, 95% CI [0.94,3.18] compared to OR 1.88, 95% CI [1.02,3.45] from S3 Table, Model 3).

Presence of another person in room

Twenty-one children indicated that there was another person in the room during the online study; of these, all but two indicated that the person was an adult, and all but three indicated that the person was not interacting with them.

In order to assess the potential influence of another person on the child's responses, we looked for an effect of *adult in room* on both the probability that the child answered questions in the on-line study correctly and on the number of quarters given in the DGs. There were eleven questions in the online study for which there was a single correct answer or response. These include: the age (in years) of the child at the time of the study, whether the child lived in more than one neighborhood (here, the "correct" answer is the parent's response to this question), how many balloons were in the box (for the drag and drop tutorial), how many quarters were in the quarter game; who gets the quarters in the box labeled "Other Child," whether the Other Child lives in Own Neighborhood or not, and whether the quarters can be used to get digital prizes at the end of the study. The last four questions were asked for the DG in Neighborhood X as well. In addi-

tion to these eleven questions, children twice had to follow instructions to demonstrate that they comprehended a Qualtrics user function (drag and drop and Heatmap--ie, clicking on a target), with a resulting response that was clearly either correct or incorrect. Counts of incorrect/correct responses, color-coded by the presence of a person/adult in the room, are illustrated in S2 Fig.

To investigate an effect of *adult in room* on correct responses for these thirteen questions, we used the R [3] package lme4 [10] to conduct multilevel binomial regression analyses, with varying intercepts for individual children. We used the Akaike Information Criterion (AIC) [11] to evaluate improvements in model fit. The inclusion of *adult in room* did not improve model fit (AIC of 523 compared to AIC of 522 for base model with varying intercepts for individuals), and the estimated standard error was over eighty times the point estimate for the coefficient; similarly, none of the other investigated covariates (*boy*, *age*) improved model fit. This suggests that when other people were in the room during the study, they were not influencing children's answers to these questions. However, the occurrence of incorrect responses being relatively rare (S2 Fig), we would be unable to detect a small effect.

To assess whether the presence of an adult in the room is predictive of DG giving, we added fixed-effect terms for the presence of an adult in the room to the multilevel ordered logit model previously used to assess the effect of condition on DG giving (S3 Table, Model 2). DIC for the model with *adult in room* is the same as the for the model without (607). The standard deviation for *adult in room* is over twice as large as the mean estimate for the coefficient (OR 1.58, 95% CI

[0.20, 13.33]). Thus, we do not discern a reliable effect of an adult's presence during the study on DG behavior.

Years lived in neighborhood and DG

We did not see a robust effect of the number of years the child had lived in their Own Neighborhood on DG behavior (standard error for years in neighborhood is 1.73 times the size of the estimated coefficient for the ordered logit regression model); however, approximately two-thirds of participants (69) had spent at least two-thirds of their lives in their Own Neighborhood.

References

1. Sampson RJ, Raudenbush SW, Earls F. Neighborhoods and violent crime: A multilevel study of collective efficacy. Science. 1997 Aug 15;277(5328):918–24.

2. Firth D. Bias reduction of maximum likelihood estimates. Biometrika. 1993;80(1):27–38.

3. R Core Team. R: A language and environment for statistical computing. Vienna, Austria: R Foundation for Statistical Computing. Version 4.3.1 [software]. 2023. Available from: https://www.R-project.org.

4. Kosmidis I. brglm: Bias reduction in binomial-response generalized linear models. R package version 0.7.2 [software] 2021. Available from: https://github.com/ikosmidis/brglm.

5. Wickham H. ggplot2: Elegant Graphics for Data Analysis. New York, NY, US: Springer-Verlag; 2016.

6. McElreath R. Statistical Rethinking: A Bayesian Course With Examples in R and Stan. Second edition. New York, NY, US: Chapman and Hall; 2020.

7. McElreath R. rethinking: Statistical Rethinking book package. R package version 2.40 [software] 2023. Available from: https://github.com/rmcelreath/rethinking.

8. McElreath R. glmer2stan: RStan models defined by glmer formulas. R package version 0.995 [software] 2013. Available from: https://github.com/rmcelreath/glmer2stan.

9. Stan Development Team. RStan: the R interface to Stan. R package version 2.26.23 [software]. 2023. Available from: https://github.com/stan-dev/rstan.

10. Bates D, Maechler M, Bolker B, Walker S. Fitting linear mixed-effects models using lme4. J Stat Softw. 2015; 67:1-48. doi:10.18637/jss.v067.i01.

11. Akaike H. A new look at the statistical model identification. IEEE Trans Automat Contr. 1974; 19(6): 716-723. doi:10.1109/TAC.1974.1100705.
