# Supplementary figures and images for: Children adjust behavior in novel social environment to reflect local prosocial norms inferred from brief exposure
Source: PLoS One. 2025 Jul 9;20(7):e0325984. doi: 10.1371/journal.pone.0325984 (PMC12240362; doi:10.1371/journal.pone.0325984)

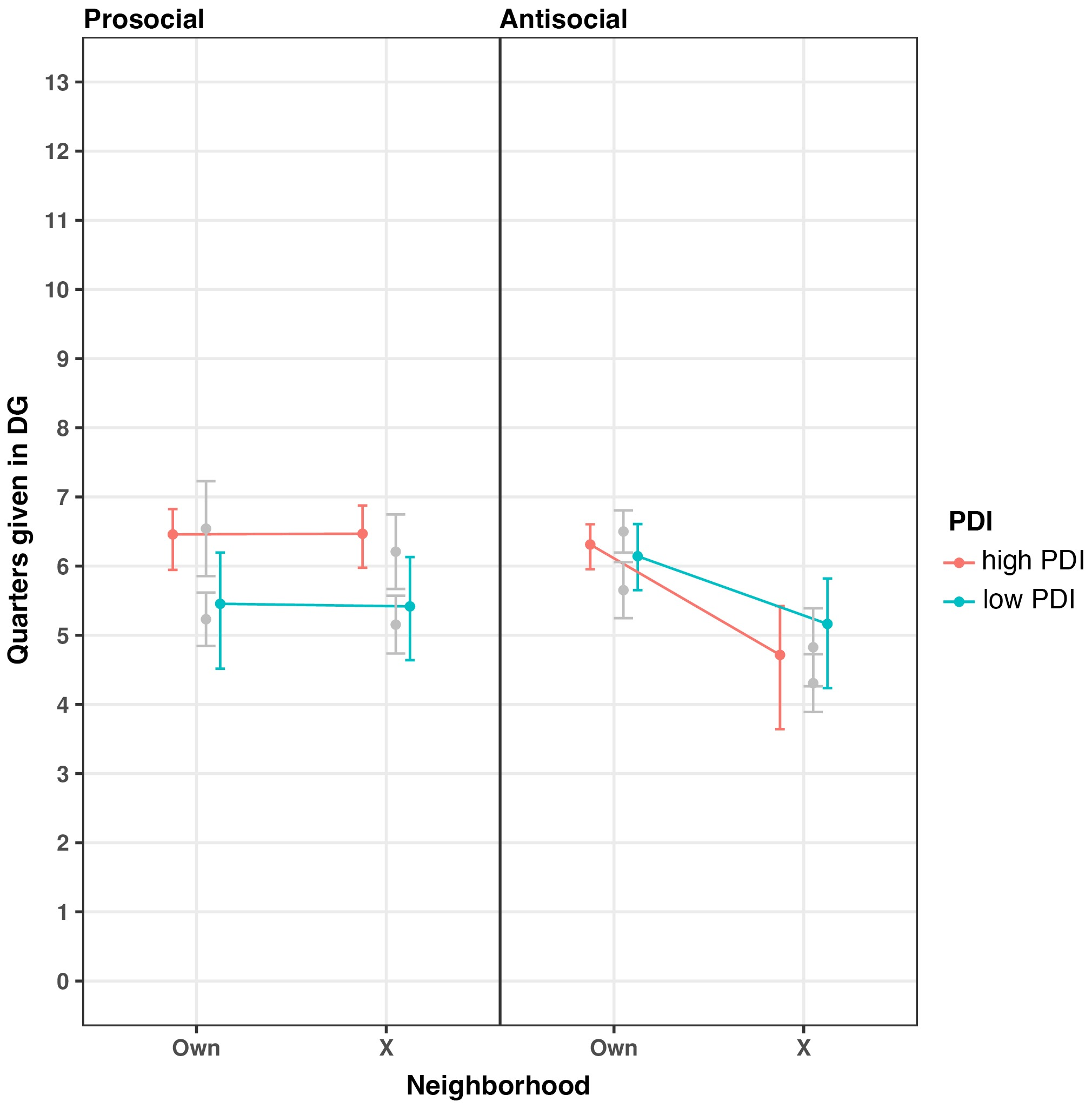

Supplement: S1 Fig — Means and standard errors of number of quarters given are plotted in gray alongside model predictions (mean and 95% CI). The model specifies an interaction among PDI, Antisocial condition, and Neighborhood X (S1 Appendix). High and low PDI represent a median split. (TIF) [file pone.0325984.s005.tif]

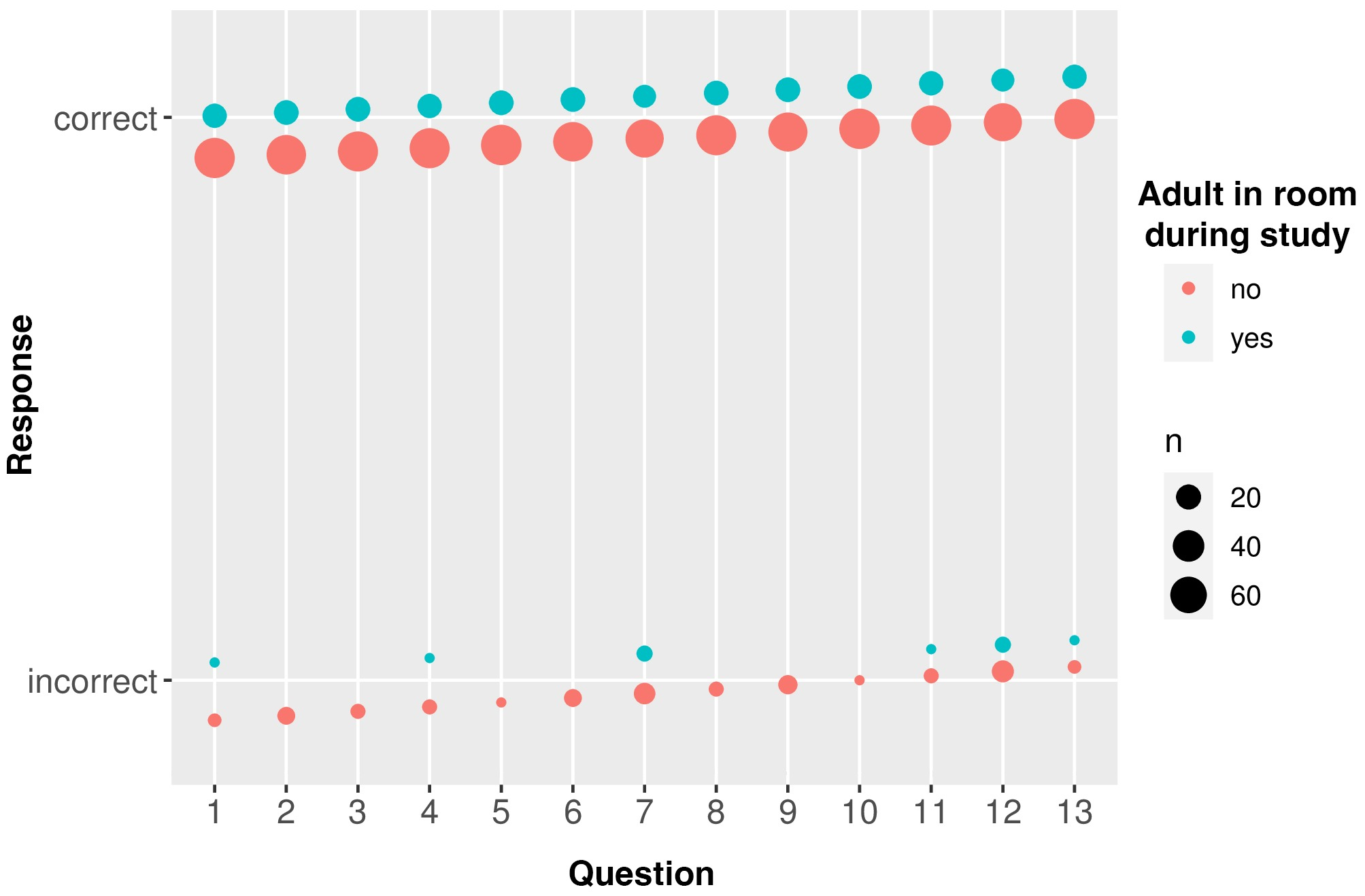

Supplement: S2 Fig — Counts of outcomes for each of 13 questions for which the response could be assigned a “correct/incorrect” value. Missing responses were treated as incorrect. As ordered, the 13 questions/instructions are: child’s age; child resides in multiple neighborhoods; successful use of drag and drop feature at first attempt during tutorial; number of balloons observed in box in during drag and drop tutorial; number of quarters in quarter game, Own Neighborhood (ON); identification (self/other) of recipient of quarters dragged to “Other Child’s Box” (ON); other child in quarter game lives in ON (true/false) (ON); quarters can be used to get digital prizes at end of study (true/false) (ON); successful clicking on target at first attempt during HeatMap tutorial; number of quarters in quarter game in Neighborhood X (NX); identification (self/other) of recipient of quarters dragged to “Other Child’s Box” (NX); other child in quarter game lives in NX (true/false) (NX); quarters can be used to get digital prizes at end of study (true/false) (NX). (TIF) [file pone.0325984.s006.tif]

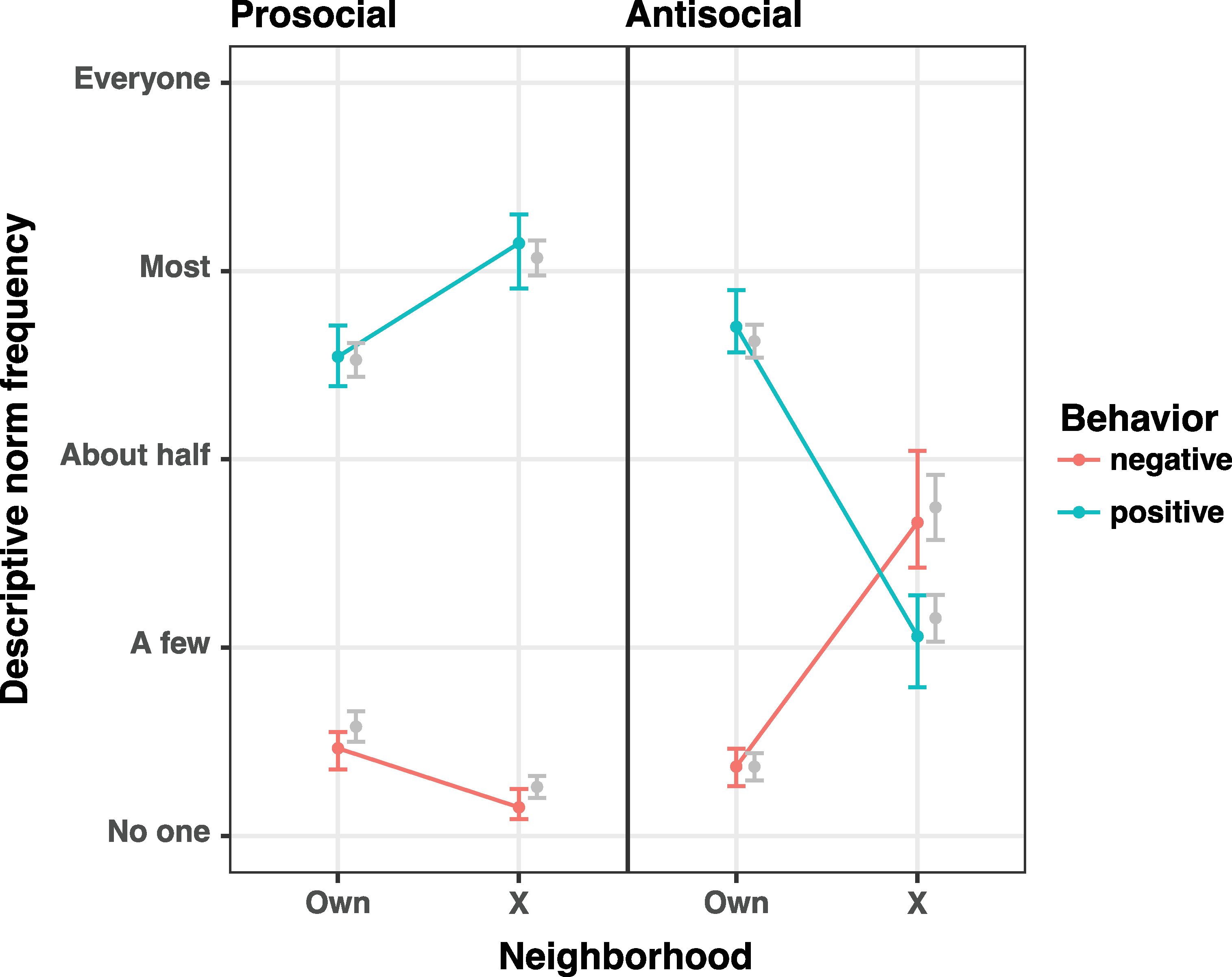

Supplement: S3 Fig — Means and standard errors of responses are plotted in gray alongside model predictions (mean and 95% CI) (see S1 Table, Model 1). (TIF) [file pone.0325984.s007.tif]
