# Supplementary material for: Children adjust behavior in novel social environment to reflect local prosocial norms inferred from brief exposure
Source: PLoS One. 2025 Jul 9;20(7):e0325984. doi: 10.1371/journal.pone.0325984 (PMC12240362; doi:10.1371/journal.pone.0325984)
Supplement: S1 Table — Ordered logit regression. Dataset includes only those questions for which there was no information in the slideshow. Predictions from Model 1 depicted in S3 Fig. Parentheses contain 95% confidence intervals for ORs. Standard deviations given for estimated variance components. “Correlation” refers to correlation between estimated slopes, intercepts. (PDF) [file pone.0325984.s008.pdf]

	Model 1			*Model 2*		
	**Estimate**	**SD**	**OR**	**Estimate**	**SD**	**OR**
**Fixed effects**						
Neighborhood X	1.69	0.38	5.42 (2.59,11.47)	-1.09	0.42	0.34 (0.15,0.79)
Antisocial condition	0.39	0.31	1.48 (0.79,2.66)			
Participant age	-0.18	0.19	0.84 (0.58,1.22)	-0.14	0.19	0.87 (0.60,1.28)
Adult actor	1.60	0.13	4.95 (3.86,6.36)	1.60	0.13	4.95 (3.80,6.49)
Negative behavior	2.98	0.17	19.69 (14.30,27.66)	2.99	0.16	19.89 (14.59,27.66)
*Neighborhood X * Antisocial*	-5.63	0.56	0.00 (0.00,0.01)			
**Variance components**						
Participant intercepts	1.24			1.24		
Slopes for Neighborhood X on Participant	2.34			3.87		
Correlation	-0.33			-0.32		
**DIC**	2334			2337		
