# Supplementary material for: Children adjust behavior in novel social environment to reflect local prosocial norms inferred from brief exposure
Source: PLoS One. 2025 Jul 9;20(7):e0325984. doi: 10.1371/journal.pone.0325984 (PMC12240362; doi:10.1371/journal.pone.0325984)
Supplement: S2 Table — Multilevel ordered logit regression. Parentheses contain either standard errors (variance components) or 95% confidence intervals (OR). Predictions from Model 2 plotted in Fig 4. (PDF) [file pone.0325984.s009.pdf]

	Model 1			Model 2			Model 3		
	**Est.**	**SD**	**OR**	**Est.**	**SD**	**OR**	**Est.**	**SD**	**OR**
**Fixed effects**									
Neighborhood X				-0.05	0.40	0.95 (0.43,2.10)	-0.70	0.52	0.50 (0.17,1.41)
Antisocial condition				0.49	0.87	1.63 (0.31,9.30)	0.45	0.889	1.57 (0.26,8.94)
Descriptive norms	0.91	0.21	2.48 (1.65,3.74)				0.63	0.31	1.88 (1.01,3.53)
Boy	-1.26	0.71	0.28 (0.07,1.12)	-1.37	0.81	0.25 (0.05,1.19)	-1.46	0.83	0.23 (0.04,1.14)
*Neighborhood X * Antisocial condition*				-3.04	0.65	0.05 (0.01,0.17)	-1.82	0.88	0.16 (0.27,0.90)
**Variance components**									
Participant intercepts	3.17 (0.44)			3.68 (0.51)			3.83 (0.53)		
**DIC**	645			607			603		
