# Supplementary material for: Children adjust behavior in novel social environment to reflect local prosocial norms inferred from brief exposure
Source: PLoS One. 2025 Jul 9;20(7):e0325984. doi: 10.1371/journal.pone.0325984 (PMC12240362; doi:10.1371/journal.pone.0325984)
Supplement: S3 Table — Multilevel binomial logistic regression. Parentheses contain either standard errors (variance components) or 95% confidence intervals (OR). (PDF) [file pone.0325984.s010.pdf]

	Estimate	SD	OR
**Fixed effects**			
Intercept	2.16	0.97	8.67 (1.63,75.94)
Neighborhood X	0.62	0.29	1.86 (1.07,3.35)
Antisocial condition	0.02	0.41	1.02 (0.45,2.34)
*Neighborhood X * Antisocial condition*	-0.45	0.40	0.64 (0.29,1.38)
**Variance components**			
Participant intercepts	1.45 (0.22)		
Behavior intercepts	1.59 (1.47)		
